# Adaptive divergence, historical population dynamics, and simulation of suitable distributions for *Picea Meyeri* and *P. Mongolica* at the whole-genome level

**DOI:** 10.1186/s12870-024-05166-6

**Published:** 2024-05-30

**Authors:** Yifu Liu, Wenfa Xiao, Fude Wang, Ya Wang, Yao Dong, Wen Nie, Cancan Tan, Sanping An, Ermei Chang, Zeping Jiang, Junhui Wang, Zirui Jia

**Affiliations:** 1 Key Laboratory of Forest Ecology and Environment of National Forestry and Grassland Administration, Ecology and Nature Conservation Institute, Chinese Academy of Forestry, Beijing, 100091 China; 2 Heilongjiang Forestry Research Institute, Harbin, 150080 China; 3 State Key Laboratory of Tree Genetics and Breeding, Research Institute of Forestry, Chinese Academy of Forestry, Beijing, 100091 China; 4 Research Institute of Forestry of Xiaolong Mountain, Gansu Provincial Key Laboratory of Secondary Forest Cultivation, Tianshui, 741022 China

**Keywords:** Adaptive differentiation, Gene flow, Selective sweeps, Climate change, Spruce

## Abstract

The taxonomic classification of *Picea meyeri* and *P. mongolica* has long been controversial. To investigate the genetic relatedness, evolutionary history, and population history dynamics of these species, genotyping-by-sequencing (GBS) technology was utilized to acquire whole-genome single nucleotide polymorphism (SNP) markers, which were subsequently used to assess population structure, population dynamics, and adaptive differentiation. Phylogenetic and population structural analyses at the genomic level indicated that although the ancestor of *P. mongolica* was a hybrid of *P. meyeri* and *P. koraiensis*, *P. mongolica* is an independent *Picea* species. Additionally, *P. mongolica* is more closely related to *P. meyeri* than to *P. koraiensis*, which is consistent with its geographic distribution. There were up to eight instances of interspecific and intraspecific gene flow between *P. meyeri* and *P. mongolica*. The *P. meyeri* and *P. mongolica* effective population sizes generally decreased, and Maxent modeling revealed that from the Last Glacial Maximum (LGM) to the present, their habitat areas decreased initially and then increased. However, under future climate scenarios, the habitat areas of both species were projected to decrease, especially under high-emission scenarios, which would place *P. mongolica* at risk of extinction and in urgent need of protection. Local adaptation has promoted differentiation between *P. meyeri* and *P. mongolica*. Genotype‒environment association analysis revealed 96,543 SNPs associated with environmental factors, mainly related to plant adaptations to moisture and temperature. Selective sweeps revealed that the selected genes among *P. meyeri*, *P. mongolica* and *P. koraiensis* are primarily associated in vascular plants with flowering, fruit development, and stress resistance. This research enhances our understanding of *Picea* species classification and provides a basis for future genetic improvement and species conservation efforts.

**Supplementary Information:**

The online version contains supplementary material available at 10.1186/s12870-024-05166-6.

## Introduction

Plants have extensive capacities for adaptation to local environments [1], and positive selection is a key driver of this local adaptation, leaving traces of selective sweeps on nearby genes linked to those that are selected [2]. Ecological specialization through local adaptation can lead to the emergence of new species, and this ecologically adaptive differentiation has been an important mechanism underlying the emergence of many species [3, 4]. In heterogeneous environments, locally specific climate variables can exert selection pressure on plants, driving adaptive differentiation [5]. However, many species in nature are still undergoing differentiation [6, 7]; thus, populations of the same species that are distributed in different regions are often mistakenly identified as different subspecies or species due to minor morphological differences [8]. The genus *Picea* is an important component of northern and subalpine forests, with approximately 16 species and 9 varieties in China alone [9]. Due to incomplete reproductive isolation between species, frequent gene flow, and a tendency toward reticulate evolution [10], the interspecific relationships and evolutionary history within the genus *Picea* have become complex, and the classification of some *Picea* species is controversial. Therefore, understanding gene flow between species and how the environment drives adaptive evolution is crucial for revealing the mechanisms of species differentiation and classification within the genus *Picea*.

*P. meyeri* and *P. mongolica* are unique species of the genus *Picea* that grow in China. *P. meyeri* is primarily found in locations such as Guandi Mountain, Wutai Mountain, and Wuling Mountain, while *P. mongolica* is mainly distributed in the eastern part of the Hunshandake Desert, occupying a relatively narrow range and serving as a component of rare sandy forest grasslands. Due to their similar morphologies and neighborhood distributions, these species are ideal study organisms for understanding *Picea* differentiation mechanisms. The renowned Chinese taxonomist Zheng Wanjun [11] considered the spruce forest in Bayanaobo, Inner Mongolia, to be a *P. koraiensis* forest. However, in 1983, Xu [12] argued on the basis of morphological identification and geographical distribution data that this forest was composed of *P. meyeri*. Wu [13] later compared the *Picea* species found in this forest with *P. meyeri* and *P. koraiensis* and proposed it to be a local variety of *P. meyeri*, named *P. meyeri* var. *mongolica*, which was later supported by Li et al. [14]. In 1994, Xu et al. [15], through morphological anatomy, isozyme testing, and chromosome karyotype analysis, concluded that this spruce was not a variety of *P. meyeri* but rather an independent species, *P. mongolica*. Zou et al. [16] also suggested that *P. mongolica* belongs to the *P. meyeri* series and evolved from *P. meyeri* as it adapted to local climatic conditions. With the development of molecular techniques, more scholars have begun studying *P. mongolica* from a molecular perspective. Studies based on random amplified polymorphic DNA (RAPD) and inter simple sequence repeat (ISSR) molecular markers [17, 18], genomic in situ hybridization [19], and chloroplast gene fragmentation [20] all indicate marked genetic differences between *P. mongolica* and other spruces, such as *P. meyeri*, suggesting that *P. mongolica* should be considered an independent species. Additionally, *P. mongolica* did not originate from interspecific hybridization between *P. meyeri* and *P. koraiensis* and appears to be phylogenetically closer to *P. meyeri* than to *P. koraiensis* [21]. However, the latest edition of the Flora of China (https://www.iplant.cn/) regards *P. mongolica* as a synonym of *P. meyeri*, not an independent species. Due to recent radiative differentiation and morphological convergence in *Picea*, it is challenging to accurately classify the members of the genus based on morphological evidence alone. Recent genetic studies on *P. meyeri* and *P. mongolica* have mostly focused on cytoplasmic genes or nuclear gene fragments, providing limited genetic information and failing to comprehensively elucidate the genetic differences between them. Therefore, the taxonomic status of these spruces has been a subject of controversy. In this study, *P. meyeri*, *P. mongolica*, and the close relative *P. koraiensis* were selected as the study organisms, and *P. pungens* and *P. likiangensis* were used as outgroups. Through genotyping-by-sequencing (GBS) simplified genome sequencing technology, this study aimed to identify whole-genome single-nucleotide polymorphism (SNP) markers to provide a reference for the classification of species in the genus *Picea* and reveal new insights into the adaptive differentiation of spruce species. The objective was to address the following three questions: (1) From a genomic perspective, can *P. mongolica* be considered an independent species? (2) How extensive is gene flow among *P. meyeri*, *P. mongolica*, and their close relative *P. koraiensis*, and what impact has gene flow had on speciation? (3) What is the nature of the *P. meyeri* and *P. mongolica* population demography, and how do these populations adapt to environmental selection pressures?

## Materials and methods

### Plant materials

**Fig. 1:**
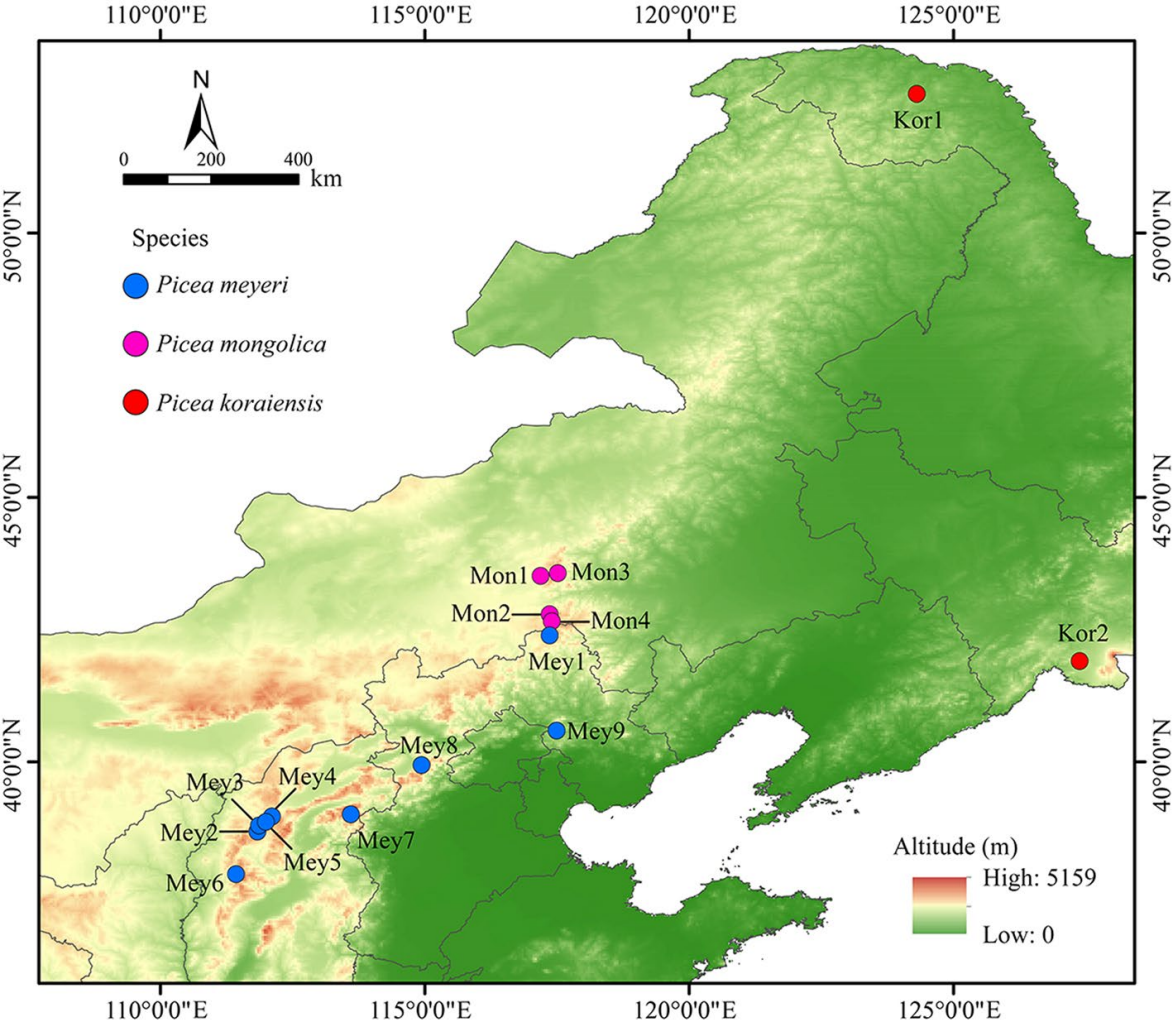
Distribution of sampling sites for *P. meyeri*, *P. mongolica* and *P. koraiensis*. Kor1 and Kor2: *P. koraiensis* from Mengke Mountain and Linjiang, respectively; Mey1, Mey2, Mey3, Mey4, Mey5, Mey6, Mey7, Mey8, and Mey9: *P. meyeri* from Saihanba, Kelan, Wuzhai, Shenchi, Ningwu, Jiaocheng, Wutai Mountain, XiaoWutai Mountain, and Wuling Mountain, respectively; Mon1, Mon2, Mon3, and Mon4: *P. mongolica* from Baiyinaobao, Dajuzi, Huanggangliang, and Huamugou, respectively

**Fig. 2:**
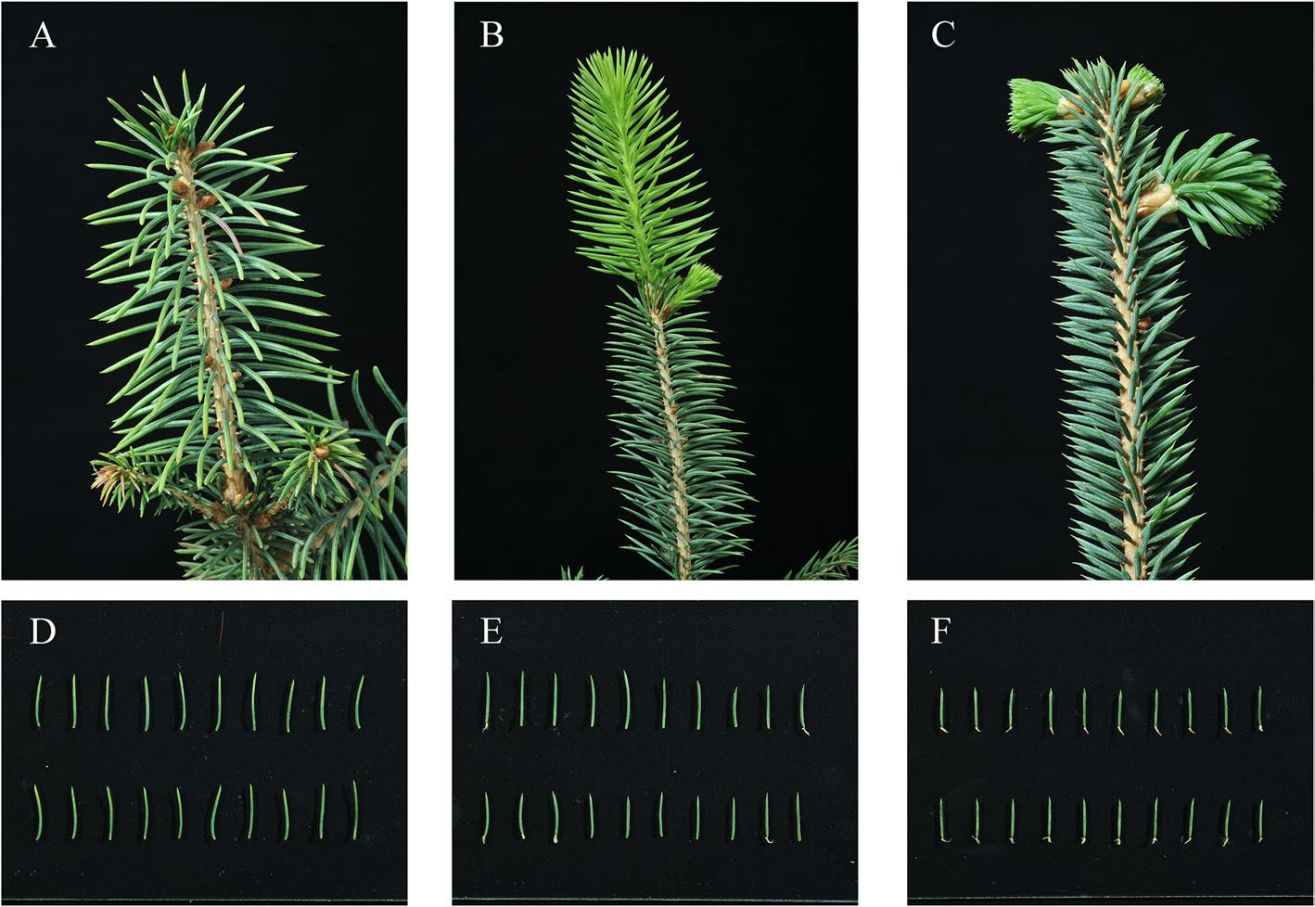
Phenotype photographs of *P. meyeri*, *P. mongolica* and *P. koraiensis*. **A,** **B** and **C** are the branches of *P. meyeri*, *P. mongolica* and *P. koraiensis*, respectively; **D**, **E** and **F** are the leaves of *P. meyeri*, *P. mongolica* and *P. koraiensis*, respectively

In this study, a total of 223 leaf samples were collected from five species within the genus *Picea*. The samples were obtained from 130 individuals belonging to 9 natural populations of *P. meyeri* and 58 individuals belonging to 4 natural populations of *P. mongolica*, covering most of the distribution areas of the two species. Additionally, the study included 25 individuals from 2 natural populations of *P. koraiensis*, 5 individuals from 1 natural population of *P. likiangensis*, and 5 individuals from 1 seed garden population of *P. pungens* (Fig. 1, Table S1). The leaves of *P. meyeri*, *P. mongolica*, and *P. koraiensis* are all quadrangular-linear. The apexes of the *P. meyeri* and *P. mongolica* leaves are acute, while those of the *P. koraiensis* leaves are obtuse. The leaves of *P. mongolica* are covered with a white powder that fills the stomatal lines, which are less pronounced in *P. meyeri* and *P. koraiensis*. The number of stomatal lines on the leaves decrease in the following order: *P. mongolica*, *P. meyeri*, and *P. koraiensis*. The cones of *P. mongolica* are larger than those of *P. meyeri* and *P. koraiensis* [15] (Fig. 2). The individuals sampled in each natural population were distributed approximately 100 m apart. The collected leaves were immediately dried with silica gel and stored at low temperature. The longitude, latitude, and altitude of each population were recorded using an eTrex handheld GPS device (Garmin, Germany). All these samples were identified by Junhui Wang, who is a professor at the Research Institute of Forestry, Chinese Academy of Forestry. All voucher specimens were deposited at the Research Institute of Forestry, Chinese Academy of Forestry, and detailed sample information is provided in Table S1.

### DNA extraction and sequencing and SNP calling

In this study, DNA was extracted from leaves using a modified cetyltrimethylammonium bromide (CTAB) method [22]. The concentration and integrity of the DNA were determined using a NanoDrop 1000 spectrophotometer (Nanodrop, MA, USA) and 1% agarose gel electrophoresis, respectively. Following DNA extraction, sequencing was performed using the double-digest GBS technique [23, 24]. For each sample, after extracting 1.5 µg of DNA, digestion was initially conducted with the *EcoRI* and *NiaIII* restriction enzymes, followed by the ligation of adaptors to both ends of the DNA fragments using T4 DNA ligase (NEB) and subsequent amplification. DNA fragments ranging from 400 to 600 bp were then recovered and purified using 1% agarose gel electrophoresis. Finally, the purified products were sequenced on the Illumina HiSeq 4000 sequencing platform (Illumina, CA, USA) using 150-bp paired-end sequencing.

For SNP calling, the raw data were filtered using Fastp v0.23.4 [25], in which the 5’-end adaptor sequences, anomalous nucleotide bases, and low-quality ends of the reads were removed (reads with ≥ 10% identified nucleotides and reads with > 50% bases having Phred quality scores ≤ 10). We aligned the filtered clean data for each *Picea* sample to the *P. abies* genome using Burrows–Wheeler Aligner (BWA) v0.7.8-r45 [26]. SNP calling was then performed using the Bayesian method in Genome Analysis Toolkit (GATK) v4 [27], followed by preliminary filtering of SNPs based on variant components to generate the SNP dataset. For subsequent phylogenetic and population analyses, the SNP dataset was filtered using VCFtools v0.1.11 [28] (minimum allele frequency ≥ 0.02, maximum missing rate ≤ 0.5) to obtain high-quality SNPs (Dataset I).

### Phylogenetic and population structural analyses

Using the whole-genome SNP dataset (Dataset I), a maximum likelihood (ML) tree was constructed in IQ-TREE v1.6.10 [29] to explore the phylogenetic relationships among species, setting the number of bootstrap iterations to 1000. Based on the Bayesian information criterion (BIC), the optimal phylogenetic tree model was selected using ModelFinder in IQ-TREE v1.6.10 [29], with the GTR + I model identified as the best model. However, considering that phylogenetic tree reconstruction was based on SNPs, the GTR + I + ASC model was ultimately chosen. The phylogenetic tree results were visualized using iTOL v5 [30]. Admixture v1.3.0 [31] was used to assess population structure. The SNP data (Dataset I) were formatted and pruned for linkage disequilibrium using VCFtools v0.1.11 and PLINK v1.90 (indep-pairwise 50 10 0.1). The number of population clusters (K) ranged from 2 to 10, with 1000 bootstrap replicates employed. The individual genetic composition coefficient (Q) of each group was used to construct a population genetic structure matrix, and cross-validation (CV) error was employed to explore convergence and determine the optimal number of clusters. The population genetic structure matrix was visualized using the Pophelper v2.3.1 package in R v4.3.1. Population genetic structure was analyzed using principal component analysis (PCA) with GCTA v1.93.3 [32] based on SNPs (Dataset I). The results were visualized using the ggplot2 v3.4.3 package in R v4.3.1. Nucleotide diversity (*π*), Tajima’s D, and population genetic differentiation (*F*_ST_) were calculated with the PopGenome v2.7.5 package in R v4.3.1. The observed heterozygosity (*H*_*o*_) and expected heterozygosity (*H*_*e*_) were computed using PLINK v1.90 with the default settings. Visualization of the PCA and *F*_ST_ results was conducted using the ggplot2 v3.4.3 package in R v4.3.1.

### Gene flow

To detect gene flow among *P. meyeri*, *P. mongolica*, and *P. koraiensis*, TreeMix v1.13 [33] and Dsuite v0.5-r44 [34] were used for TreeMix analysis and ABBA–BABA analysis, respectively. To minimize the effects of linkage disequilibrium, SNPs (Dataset I) were filtered (indep-pairwise 50 10 0.1) using PLINK v1.90 [35] to obtain Dataset II for gene flow analysis. In the TreeMix analysis, the migration parameter (m) values were set from 1 to 10, with 5 repetitions for each m value. We utilized the OptM v0.1.6 package in R v4.3.1 to determine the optimal number of migration edges. Subsequently, visualization was conducted using the built-in R script “plotting_funcs.R” in TreeMix v1.13. In the ABBA–BABA analysis, Patterson’s *D*-statistic was computed using the Dtrios program from Dsuite v0.5-r44. *P. pungens* and *P. likiangensis* were used as outgroups (O), *P. koraiensis* was designated the ancestral group (P1), *P. meyeri* was designated sister group I (P2), and *P. mongolica* was designated sister group II (P3). In summary, for the {O, [P1, (P2, P3)]} combination, the ABBA site pattern refers to the sharing of derived alleles between P1 and P2, while the BABA site pattern refers to the sharing of derived alleles between P1 and P3. Under the null hypothesis of incomplete lineage sorting, the numbers of ABBA and BABA site patterns should be roughly equal (D = 0). A significant deviation of D from 0 indicates the occurrence of gene flow events. The significance of the ABBA–BABA result was determined by using a jackknife method to calculate the Z score (Z score > 3).

### Population dynamics analysis

Historical population dynamics were analyzed using Stairway Plot v2.2.1 [36] based on the site frequency spectrum (SFS) of SNPs (Dataset I), where the SFS was computed using the Python v3.9 script easySFS (https://github.com/isaacovercast/easySFS). The SFS state was designated “folded” to calculate the SFS of the second allele. Projection values with as many selected loci as possible were chosen to output SFS information. The SFS information was input into the required blueprint file. The generation time was set at 50 years, and the per-generation neutral mutation rate was set at 2.5 × 10^−8^ [37, 38]. Moreover, the number of randomly selected loci was set to 67%, and 200 inputs were used to estimate the median *N*_*e*_ and 95% pseudoconfidence interval (CI).

### Species distribution model (SDM)

The point data for *P. meyeri* and *P. mongolica* distribution were primarily sourced from the Chinese Virtual Herbarium (CVH, http://www.cvh.ac.cn/), the National Specimen Information Infrastructure of China (http://www.nsii.org.cn), and published literature [39]. After filtering through ENM Tools v1.4, 77 effective distribution points for *P. meyeri* and 31 points for *P. mongolica* were retained (Table S2). The data for nineteen environmental factors were downloaded from the WorldClim database (http://www.worldclim.org), with a resolution of 2.5 arc min. To avoid overfitting the species distribution models (SDMs) due to multicollinearity, Pearson correlation coefficients between the environmental factors were calculated; for variables with |r| > 0.7, the variable with the smaller contribution (estimated through a jackknife test) was removed. The Kuenm v1.1.3 package in R v3.6.3 was used to select the best feature types and regularization multipliers. The Maxent v3.4.4 model was applied to run the SDMs for the two *Picea* species during four periods (the Last Glacial Maximum (LGM), mid-Holocene (MH), present day and 2070s) using the CCSM4 atmospheric circulation model. In the Maxent model, 30% of the distribution data were selected as the test dataset, and the rest of the data were selected as the training dataset. The maximum number of iterations was set to 5000 to ensure that the model had enough time to converge. Finally, the SDM results were visualized, and the suitable habitat areas were inferred using ArcGIS v10.7.

### Environmental association analysis

#### Selection of environmental variables

The present-day data for the 19 environmental factors at each *P. meyeri* and *P. mongolica* distribution point were extracted using ArcGIS v10.7 (Table S2). The Pearson correlation coefficients for the bioclimatic variables were calculated using the ‘cor’ function in R v4.3.1, retaining only one variable for pairs with |r| > 0.7. Additionally, the variance inflation factor (VIF) for each variable was calculated with the vegan v2.6.4 package in R v4.3.1 to evaluate multicollinearity, ensuring that the retained variables had a VIF < 10. Finally, on the basis of the Pearson correlation coefficients and the VIFs, four variables (BIO01: annual mean temperature; BIO03: isothermality; BIO12: annual precipitation, and BIO15: precipitation seasonality) were used for the combined *P. meyeri* and *P. mongolica* analysis.

#### Isolation by environment (IBE) and isolation by distance (IBD)

The genetic distances (*F*_ST_/(1-*F*_ST_)) for pairs of species were calculated based on the SNPs (Dataset I) using VCFtools v0.1.11. Environmental distances, representing isolation by environment (IBE), were computed using Euclidean distances with the R v4.3.1 package vegan v2.6.4. Geographical distances, representing isolation by distance (IBD), were calculated using Geodesic with the R v4.3.1 package geodist v0.08. To disentangle the effects of IBD and IBE, the partial Mantel test with the R package vegan v2.6.4 was used to quantify IBD while controlling for environmental effects and to quantify IBE while controlling for geographical effects. The significance of the partial Mantel tests was assessed through 999 permutations.

#### Redundancy analysis (RDA)

An RDA of genetic variation (SNPs, Dataset I) was conducted using the vegan v2.6.4 package in R v4.3.1 to assess the impact of environmental factors, with affected SNPs identified based on a *p* value threshold (*p* < 0.05). These SNPs were subsequently mapped onto the reference genome of *P. abies* to identify genes influenced by environmental factors. The candidate genes were subjected to Gene Ontology (GO) analysis via the OMICSHARE cloud platform (http://www.omicshare.com/tools/). Additionally, BLASTX was used to compare these genes with the *Arabidopsis thaliana* proteome to elucidate protein function information, with an E-value threshold set at 1e-5.

### Selective sweep analyses

To detect regions under significant selective sweeps in *P. meyeri* and *P. mongolica*, we used *P. koraiensis* as a background group based on the phylogenetic tree results and compared *P. meyeri* and *P. mongolica* separately with this background group to identify regions under selection. Additionally, we compared the selected regions between *P. mongolica* and *P. meyeri*. We selected windows with significantly high *π* ratios (top 5%) and significantly high *F*_ST_ values (top 5%) as regions showing significant signals of selective sweeps. This was accomplished using VCFtools v0.1.11 to compute *π* and *F*_ST_ based on Dataset I with a sliding window approach (10,000-kb windows sliding in 1000-kb steps). Additionally, we conducted Gene Ontology (GO) enrichment analysis and Kyoto Encyclopedia of Genes and Genomes (KEGG) pathway enrichment analysis on the genes within these selected regions using the OMICSHARE cloud platform (http://www.omicshare.com/tools/) with an E-value threshold set at 1e-5. This enabled us to understand the biological functions of the selected genes.

## Results

### Sequencing quality

A total of 223 *Picea* samples were sequenced using GBS, yielding 1182.66 Gb of raw data. After filtering, 1138.58 Gb of clean data were obtained, with an average of 5.11 Gb of clean data per sample. The sequencing quality was high, with Q20 > 97.27% and Q30 > 92.71%. A total of 5,716,899 raw SNPs were called, and filtering yielded 1,290,066 high-quality SNPs.

### Phylogenetic and population structure analyses

**Fig. 3:**
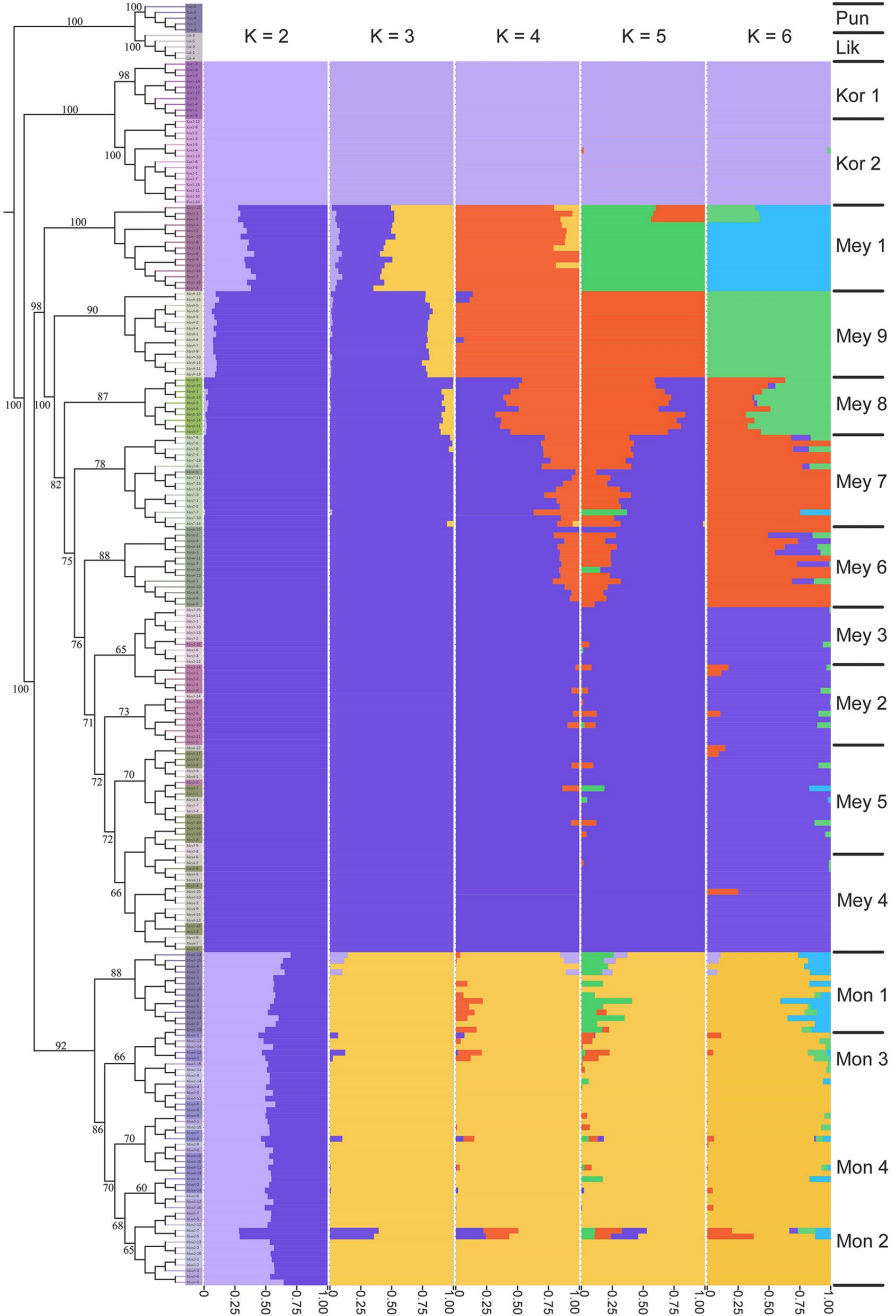
ML phylogenetic tree and structure histogram based on SNP markers. Pun: *P. pungens*; Lik: *P. likiangensis*. The remaining species abbreviations are the same as those in Fig. 1

**Fig. 4:**
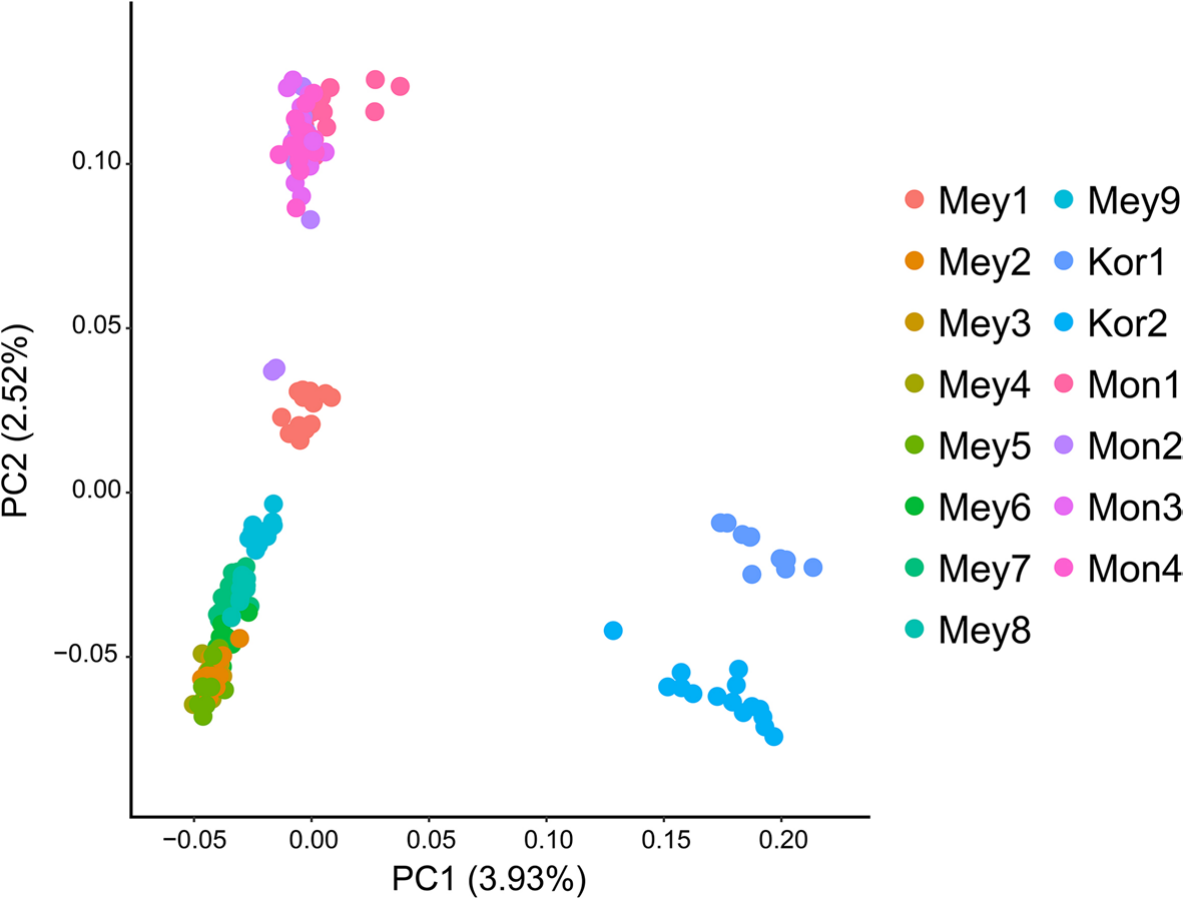
Principal component analysis of *Picea* populations. The species abbreviations are the same as those in Fig. 1

The ML phylogenetic tree (Fig. 3) showed that by using *P. pungens* and *P. likiangensis* as outgroups, the 15 populations could be divided into three clades. The first clade consisted of two populations of *P. koraiensis*, the second clade comprised nine populations of *P. meyeri*, and the third clade encompassed four populations of *P. mongolica*. Among the *P. meyeri* populations, three collected from Hebei Province were distinguishable from six collected from Shanxi Province. Notably, the four populations from the Luya Mountain area in Xinzhou, Shanxi, were closely related. Among the *P. mongolica* populations, the Baiyinaobao population differed from the other three groups. Furthermore, we evaluated the population structure of *P. koraiensis*, *P. meyeri*, and *P. mongolica* under different *K* values (Fig. 3), and the CV error (Fig. S1) decreased when *K* ranged from 2 to 6. At *K* = 2, *P. koraiensis* and *P. meyeri* formed distinct genetic components, with *P. mongolica* being composed of two genetic components. As *K* increased, *P. mongolica* differentiated from the geographic populations of *P. meyeri* earlier to form an independent genetic component, suggesting significant differences between *P. mongolica* and the various *P. meyeri* geographic populations. At *K* = 4, two *P. meyeri* populations near the *P. mongolica* distribution area differentiated earlier to form an independent genetic component. At *K* = 6, the four populations from the Luya Mountain area clustered into one genetic component, consistent with the evolutionary tree results. To understand the population structure characteristics, we also used principal component analysis (PCA) to reveal the relationships among populations. As shown in Fig. 4, P. *koraiensis*, *P. meyeri*, and *P. mongolica* were distinguishable, and *P. mongolica* was more closely related to *P. meyeri*, with the classification results being consistent with the evolutionary tree and structural analysis findings.

### Population diversity and genetic divergence

**Fig. 5:**
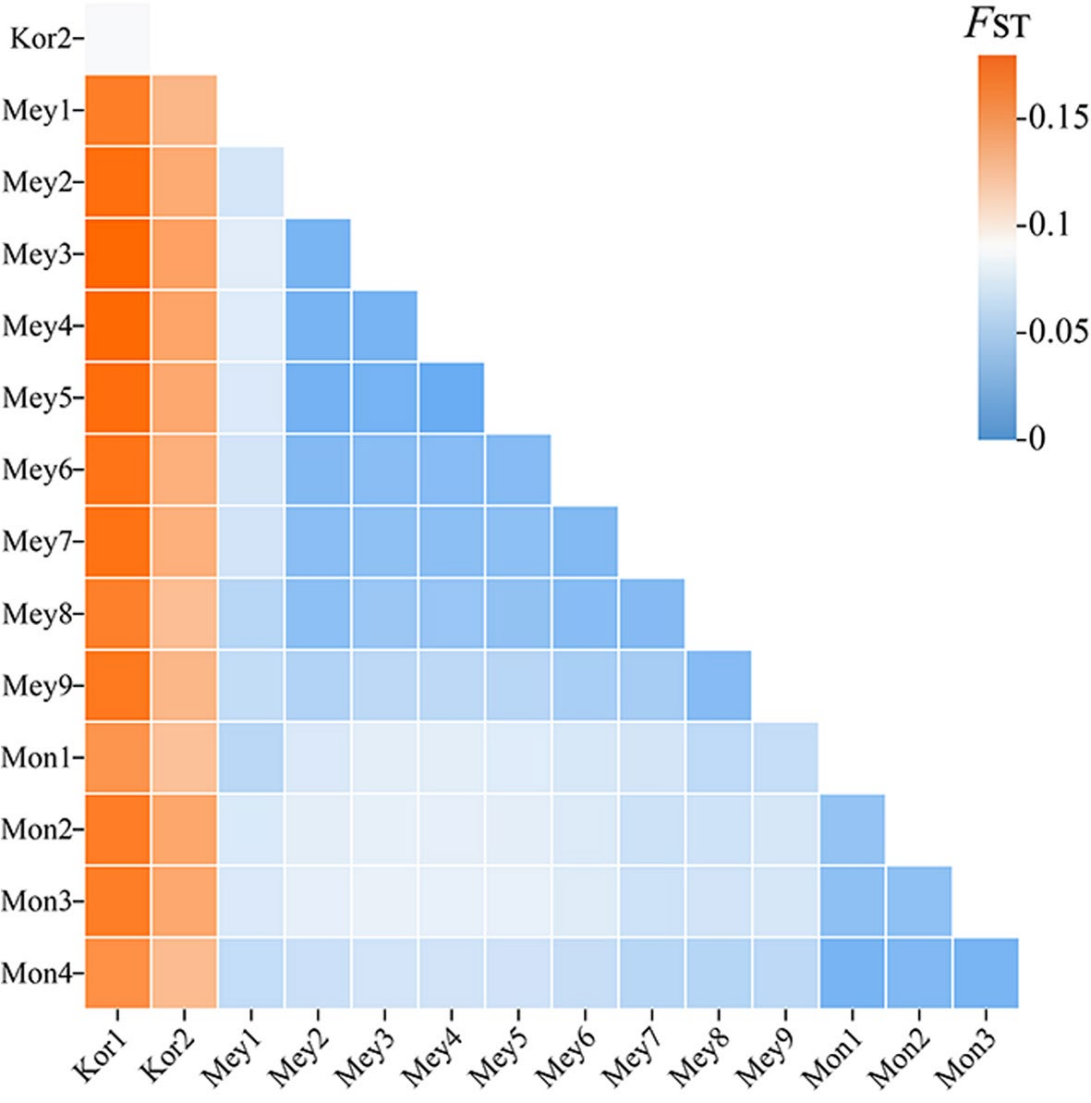
Interspecific genetic differentiation of *P. koraiensis*, *P. meyeri* and *P. mongolica*. The species abbreviations are the same as those in Fig. 1

Overall, based on the *π* values calculated from the whole-genome SNPs (Table 1), the nucleotide diversity of *P. mongolica* was greater than that of *P. meyeri* and *P. koraiensis*. The *P. mongolica* population Mon1 had the highest *π* value of 1.5009 × 10^−5^, followed by the Mon4 population, with a *π* value of 1.4968 × 10^−5^. The lowest *π* value was found for the *P. koraiensis* population Kor1, at 1.3156 × 10^−5^. The observed heterozygosity (*H*_*o*_) of the *P. koraiensis*, *P. meyeri* and *P. mongolica* populations were all lower than the expected heterozygosity (*H*_*e*_). The *H*_*o*_ and *H*_*e*_ of the *P. mongolica* populations Mon1 and Mon4 were greater than those of the other populations. The lowest *H*_*e*_ of 0.1810 was observed for the *P. koraiensis* population Kor1, and the *P. mongolica* population Mon2 had the lowest *H*_*o*_ of 0.1422. The Tajima’s D values for all 15 populations of the three species were greater than 0, suggesting that these species had rare alleles at low frequencies and likely underwent population contraction. Overall, the Tajima’s D value for *P. mongolica* was lower than that for *P. meyeri* and *P. koraiensis*. The highest Tajima’s D value was found for the *P. koraiensis* population Kor2, at 0.5857, followed by the Kor1 population, at 0.5420. The *P. mongolica* population Mon4 had the lowest value, at 0.3945. As indicated in Fig. 5, the interspecific differences between *P. koraiensis* and the other two species, *P. meyeri* and *P. mongolica*, were significant, indicating a greater degree of differentiation for *P. koraiensis*. The intraspecific differences within *P. meyeri* and *P. mongolica* were smaller than the interspecific differences between them, which was consistent with the results of the phylogenetic and population structure analyses.

**Table 1:** Estimates of genetic variation in *P. koraiensis*, *P. meyeri* and *P. mongolica*

Population	π	H_e_	H_o_	Tajima’s D
Kor1	1.3156 × 10^−5^	0.1810	0.1517	0.5420
Kor2	1.3931 × 10^−5^	0.1959	0.1701	0.5857
Mey1	1.3470 × 10^−5^	0.1904	0.1553	0.4850
Mey2	1.3729 × 10^−5^	0.1930	0.1603	0.5139
Mey3	1.3456 × 10^−5^	0.1882	0.1469	0.5211
Mey4	1.3734 × 10^−5^	0.1929	0.1613	0.5270
Mey5	1.3880 × 10^−5^	0.1950	0.1654	0.5413
Mey6	1.4019 × 10^−5^	0.1973	0.1741	0.5274
Mey7	1.3905 × 10^−5^	0.1943	0.1668	0.4686
Mey8	1.4297 × 10^−5^	0.1980	0.1880	0.4661
Mey9	1.3766 × 10^−5^	0.1936	0.1682	0.5411
Mon1	1.5009 × 10^−5^	0.2102	0.1821	0.4068
Mon2	1.4335 × 10^−5^	0.1973	0.1422	0.4536
Mon3	1.4486 × 10^−5^	0.2008	0.1506	0.4609
Mon4	1.4968 × 10^−5^	0.2103	0.1748	0.3945

The species abbreviations are the same as in Fig. 2

### Gene flow

**Fig. 6:**
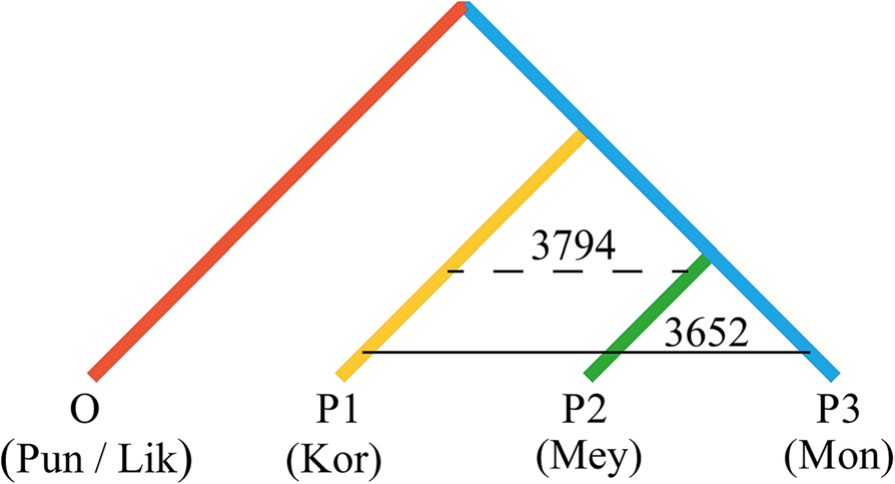
ABBA–BABA test tree models for *P. koraiensis*, *P. meyeri* and *P. mongolica*. Pun: *P. pungens*; Lik: *P. likiangensis*; Kor: *P. koraiensis*; Mey: *P. meyeri*; Mon: *P. mongolica*

**Fig. 7:**
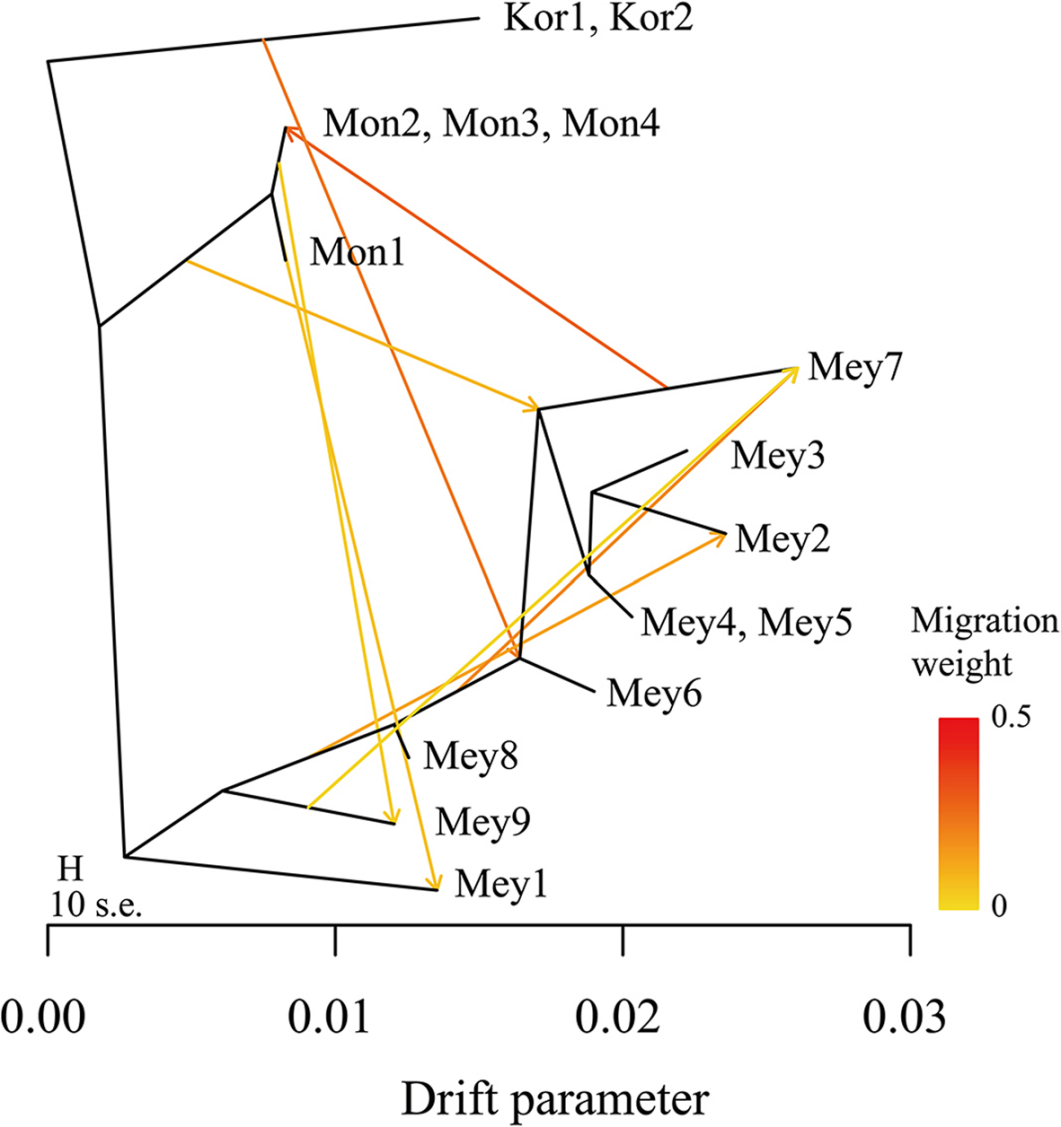
Gene flow among *P. koraiensis*, *P. meyeri* and *P. mongolica*. The species abbreviations are the same as those in Fig. 1

We first employed the ABBA–BABA method to analyze gene flow among three species: *P. koraiensis*, *P. meyeri*, and *P. mongolica*. A total of 3794 alleles were shared between P1 and P2, and 3652 alleles were shared between P1 and P3, with a D value of 0.019 and a Z score of 20.16, indicating significant asymmetrical gene flow between *P. koraiensis* and the two sister groups; that is, there was significant gene flow between *P. koraiensis* and *P. meyeri* (Fig. 6). To clarify the direction of gene flow among these three species, we utilized TreeMix analysis to construct a ML tree (Fig. 7). The optimal number of migration edges was eight (Fig. S2), suggesting eight gene flow events among the three species. Historically, there was heavy gene flow from *P. koraiensis* to *P. meyeri* but no significant gene flow from *P. koraiensis* to *P. mongolica*, consistent with the ABBA–BABA results. Additionally, we revealed a history of strong gene flow from *P. meyeri* to *P. mongolica*, three gene flow events from *P. mongolica* to *P. meyeri*, and three intraspecific gene flow episodes between *P. meyeri* populations, indicating frequent gene flow within *P. meyeri* populations and between *P. meyeri* and *P. mongolica*.

### Historical population dynamics of *P. Meyeri* and *P. Mongolica*

**Fig. 8:**
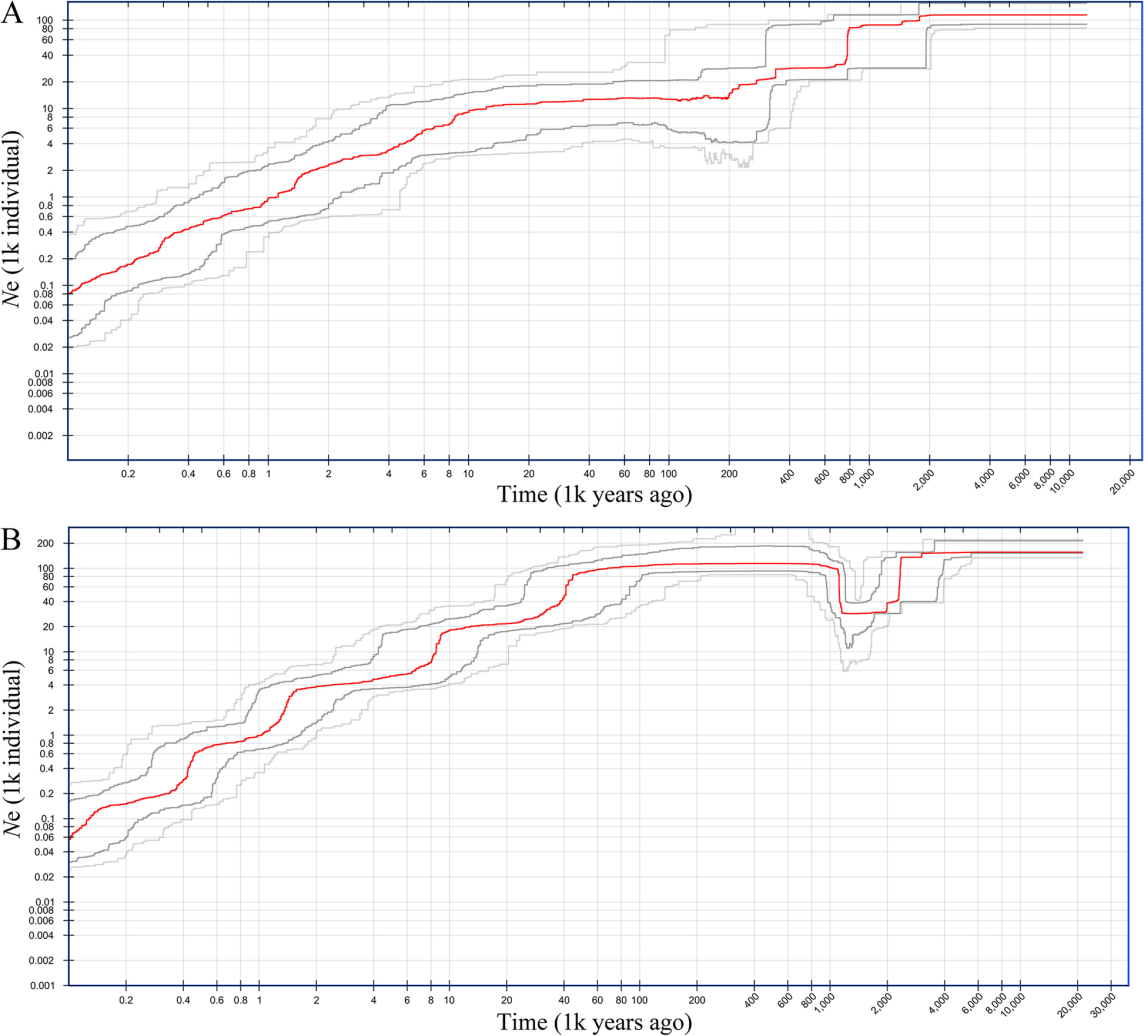
Demographic history of *P. meyeri* (**A**) and *P. mongolica* (**B**). Dark gray lines: 75% confidence intervals of the inferences. Light gray lines: 95% confidence intervals of the inferences

As illustrated in Fig. 8, the effective population size (*N*_e_) of *P. meyeri* began to gradually decline beginning in the early Pleistocene (approximately 2 Ma), followed by a period of stability. A sharp decline occurred during the middle Pleistocene (0.8 Ma), indicating a population bottleneck, after which the effective population size stabilized. *N*_e_ continued to decrease gradually from 0.38 Ma to 0.2 Ma, stabilized until 0.1 Ma, and once again started to decrease rapidly. For *P. mongolica*, *N*_e_ experienced a sharp decline during the early Pleistocene (2.3–2.0 Ma), leading to a population bottleneck, after which it began to expand at approximately 1.2 Ma, reaching a minor peak before stabilizing. Subsequently, rapid decreases occurred at 0.45 Ma and 0.09 Ma (Fig. 8). Overall, the effective population sizes of *P. meyeri* and *P. mongolica* gradually decreased.

### Changes in potential habitats

The area under the curve (AUC) values of the Maxent model for both *P. meyeri* and *P. mongolica* were greater than 0.9 (Fig. S3), indicating excellent model accuracy. Jackknife tests and the percentage contribution results (Fig. S4) showed that BIO02 (mean diurnal range), BIO04 (temperature seasonality), BIO10 (mean temperature of the warmest quarter), BIO13 (precipitation of the wettest month), BIO15 (precipitation seasonality), and BIO17 (precipitation of the driest quarter) were key environmental factors for predicting the geographic distribution of *P. meyeri*. For *P. mongolica*, the best environmental predictors were BIO03 (isothermality), BIO04, BIO09 (mean temperature of the driest quarter), BIO15, and BIO17. Thus, seasonal variations in temperature and precipitation, as well as the minimum amount of precipitation, had significant impacts on the distributions of *P. meyeri* and *P. mongolica*.

**Fig. 9:**
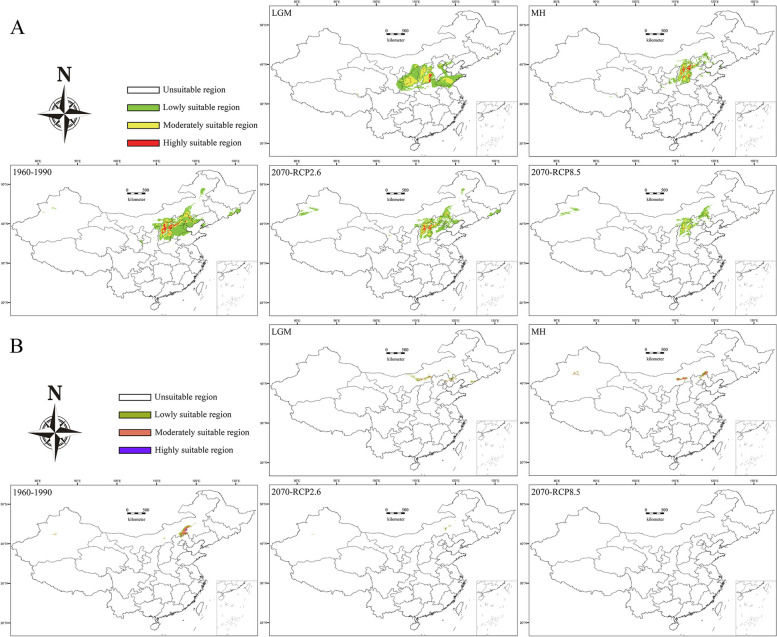
Prediction of potentially suitable habitats for *P. meyeri* (**A**) and *P. mongolica* (**B**)

The current potential habitat of *P. meyeri* is located mainly in the Lüliang, Taihang, and Yanshan Mountains, covering provinces and cities such as Shanxi, Hebei, and Beijing. The total area of the potential habitat is approximately 5.02 × 10^5^ km^2^, with 2.85 × 10^4^ km^2^ being highly suitable and 1.08 × 10^5^ km^2^ being moderately suitable. The current potential habitat of *P. mongolica* is located primarily at the eastern edge of the Hunshandake Desert, which includes parts of Inner Mongolia, with a total area of approximately 3.67 × 10^4^ km^2^, including 1.39 × 10^3^ km^2^ of highly suitable land and 1.51 × 10^4^ km^2^ of moderately suitable land. Under historical climatic conditions, the potential habitat areas of *P. meyeri* and *P. mongolica* continuously decreased from the LGM to the MH but significantly expanded from the MH to the present. The area of highly suitable habitat for *P. meyeri* and *P. mongolica* expanded from the LGM through the MH to the present. The distribution ranges of both species showed trends of moving northeastward from the LGM to the present. Simultaneously, the potential geographical distributions of the two *Picea* species under two emission scenarios for the future (2070s) were simulated. Under the RCP2.6 emission scenario, the total suitable area for *P. meyeri* would be 3.26 × 10^5^ km^2^, with a highly suitable area of 1.34 × 10^4^ km^2^, representing decreases of 35.18% and 52.80%, respectively, from the current potential distribution area. The total suitable area for *P. mongolica* would be 3.00 × 10^3^ km^2^, a decrease of 91.73% from the current potential distribution, and the highly suitable area would disappear. Under the RCP8.5 emission scenario, the total suitable area for *P. meyeri* would be 1.68 × 10^5^ km^2^, with a highly suitable area of 0.07 × 10^4^ km^2^, representing drastic decreases of 66.53% and 97.45%, respectively, from the current potential distribution, while the suitable habitat for *P. mongolica* would disappear (Fig. 9).

### Environmental adaptability and genetic variation

**Fig. 10:**
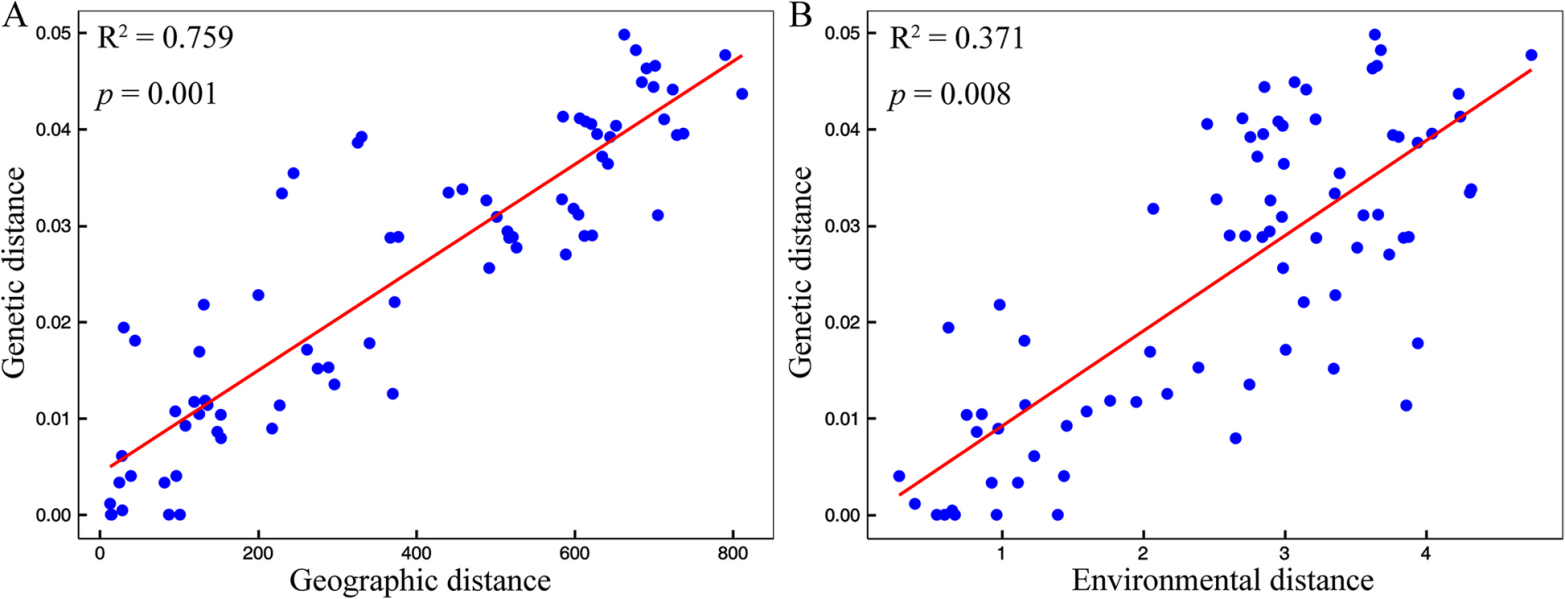
Partial Mantel tests of genetic distance against (**A**) geographical distance and (**B**) environmental distance

As illustrated in Fig. 10, genetic distance was strongly associated with environmental distance for *P. meyeri* and *P. mongolica* combined, indicating local adaptation to the environment within populations of these two spruce species. Genetic distance was also significantly associated with environmental distance based on four environmental variables. The results for IBD and IBE confirm that both geographic and environmental distances strongly influence genetic distance.

**Fig. 11:**
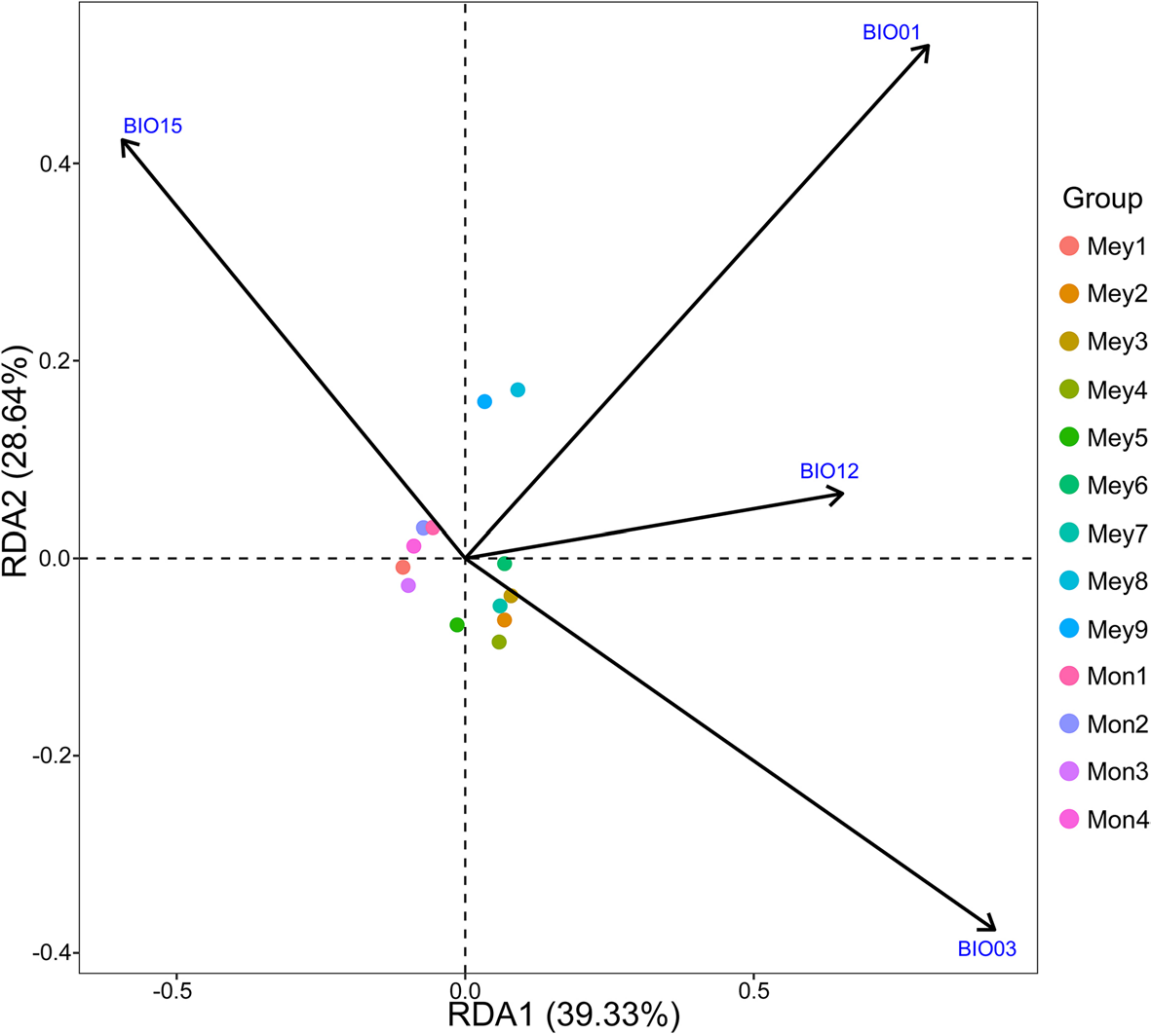
RDA of genetic variation and environmental variables. The species abbreviations are the same as those in Fig. 1

 Among the whole-genome SNPs distributed across 13 populations of *P. meyeri* and *P. mongolica*, 96,543 were associated with the environmental factors BIO01, BIO03, BIO12, and BIO15 (Table S3). Figure 11 shows that the first three axes of the RDA explained 39.33%, 28.46%, and 18.51% of the variation, respectively. BIO15 contributed most significantly to the genetic variation in the four populations of *P. mongolica* and the Mey1 population. BIO3 explained most of the genetic variation in the Mey2, Mey3, Mey4, Mey5, and Mey7 populations. BIO1 made the most significant contribution to the genetic variation in Mey8 and Mey9, while BIO03 and BIO12 explained most of the genetic variation in Mey6. The GO enrichment analysis results (Fig. S5) suggested that the potential candidate genes related to environmental variables are involved mainly in biological processes such as the response to water deprivation, response to abiotic stimuli, and response to hormones. The main molecular functions of these genes included carbohydrate derivative binding, phospholipase activity, and hydrolase activity. Using BLASTX, candidate genes were compared against the Arabidopsis protein database, revealing many proteins related to environmental adaptability (Table S4), such as proteins related to drought (e.g., PUB22/23, BAM1, and SUS3), abiotic stress (e.g., CAT2, Sect. 14, and ERD4), and disease resistance (e.g., RDR1, TAO1, and SGT1b).

### Genes experiencing selective sweeps in *P. Meyeri* and *P. Mongolica*

A total of 167 genes under selection were identified across these three comparisons. Specifically, 65 common genes under selection were identified between *P. meyeri* and the reference group (Table S5), 72 genes under selection were common between *P. mongolica* and the reference group (Table S6), and 30 genes under selection were common between *P. meyeri* and *P. mongolica* (Table S7).

**Fig. 12:**
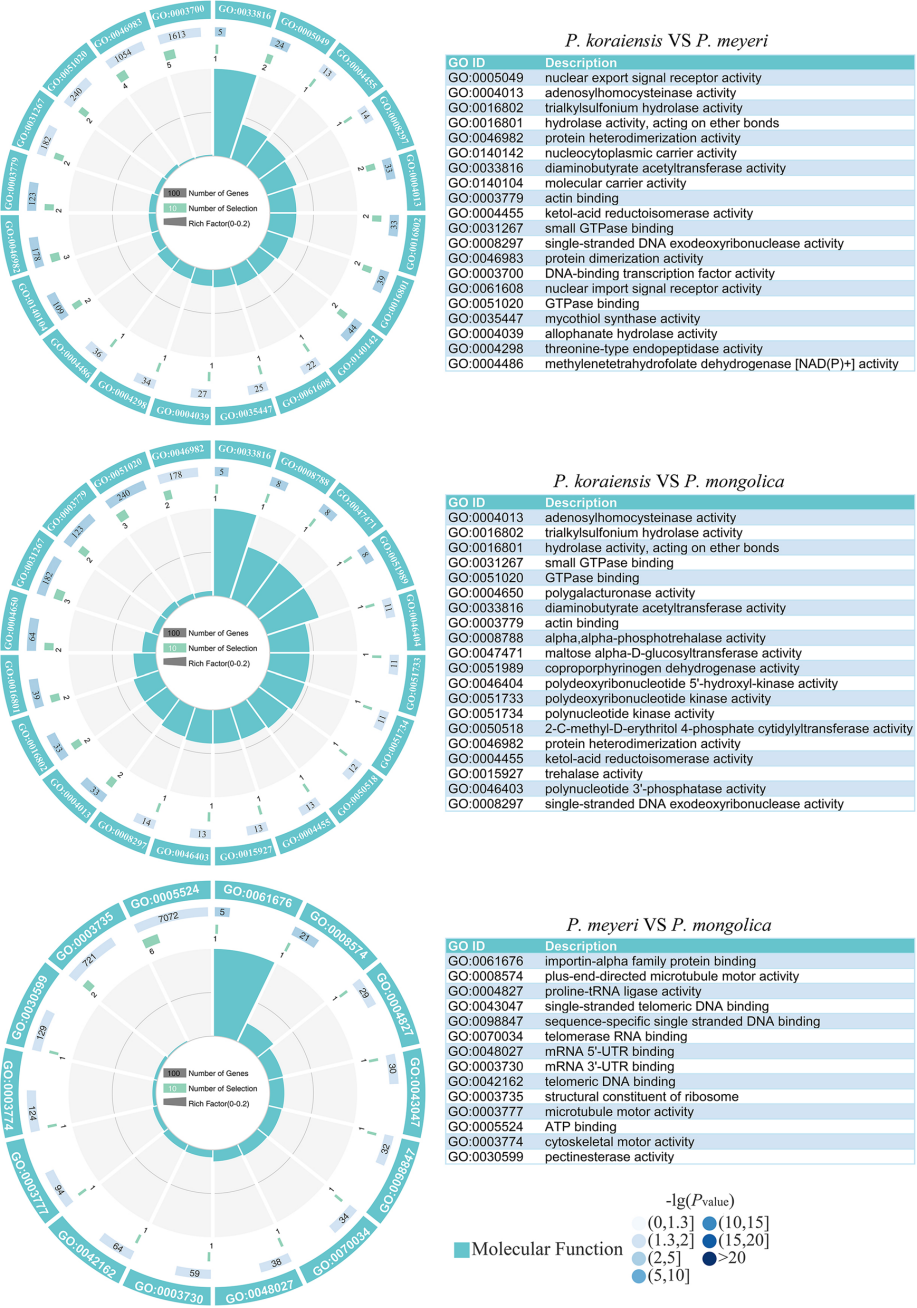
GO analysis of genes experiencing selective sweeps

Figure S6 clearly shows that the selective sweep areas between *P. meyeri* and the reference group are defined by *π*_kor_/*π*_mey_ ≥ 2.89 and *F*_ST_ ≥ 0.34. The GO enrichment analysis (Table S8) revealed that the selected genes were annotated with 202 terms, among which 145, 30, and 27 belonged to the biological process (BP), cellular component (CC), and molecular function (MF) subgroups, respectively. The MFs were mainly associated with protein heterodimerization activity, hydrolase activity acting on ether bonds, adenosylhomocysteinase activity, etc. (Fig. 12).

**Fig. 13:**
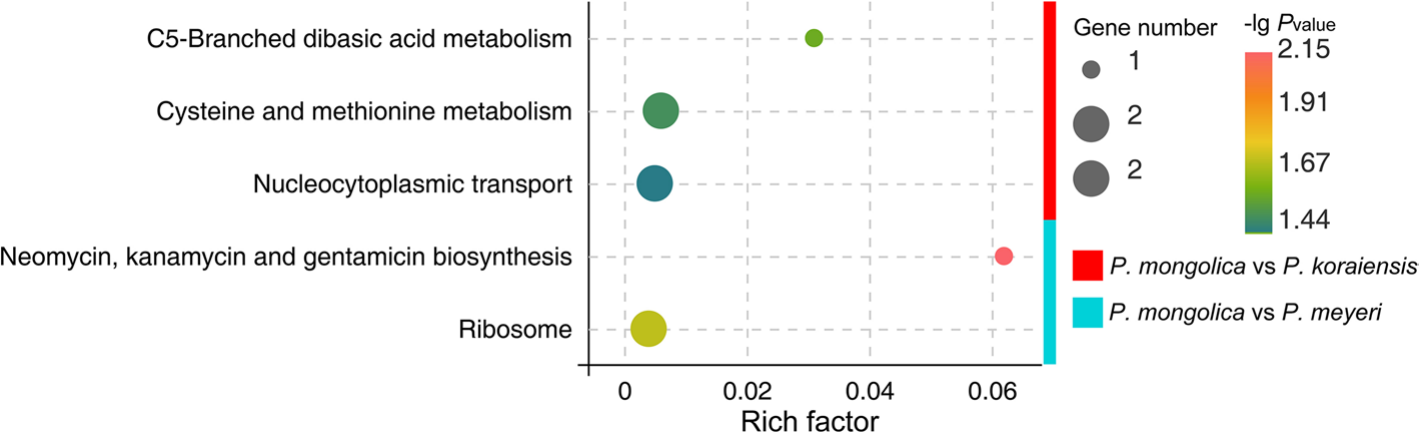
KEGG pathway analysis of genes experiencing selective sweeps

The selective sweep areas between *P. mongolica* and the reference group were defined by *π*_kor_/*π*_mon_ ≥ 2.14 and *F*_ST_ ≥ 0.33 (Fig. S6). Based on the GO enrichment analysis (Table S9), selected genes were annotated with 290 terms, with 210, 33 and 47 terms in the BP, CC, and MF subgroups, respectively. The MF terms were primarily those associated with GTPase binding, adenosylhomocysteinase activity, hydrolase activity acting on ether bonds, etc. (Fig. 12). The KEGG enrichment analysis (Fig. 13) indicated that the genes were involved mainly in pathways related to nucleocytoplasmic transport, cysteine and methionine metabolism, and C5-branched dibasic acid metabolism.

The selective sweep areas between *P. mongolica* and *P. meyeri* were defined by *π*_mey_/*π*_mon_ ≥ 1.56 and *F*_ST_ ≥ 0.14 (Fig. S6). The GO enrichment analysis (Table S10) revealed that the selected genes were annotated with 166 terms, among which 108, 44, and 14 belonged to the BP, CC, and MF subgroups, respectively. The MFs were mainly those related to ATP binding, structural constituents of ribosomes, importin-alpha family protein binding, etc. (Fig. 12). The KEGG enrichment analysis (Fig. 13) revealed that the genes were involved in pathways such as neomycin, kanamycin and gentamicin biosynthesis and ribosomes.

## Discussion

### Species differentiation and gene flow characteristics

*P. meyeri* and *P. mongolica* are unique, ecologically important spruce tree species native to China. However, the taxonomic status of *P. mongolica* has been controversial. Our study revealed that these two species form independent branches in a phylogenetic tree and that *P. mongolica* formed independent genetic clusters earlier than the geographical populations of *P. meyeri*. It also formed a distinct cluster separate from *P. meyeri* and *P. koraiensis* in the PCA. Furthermore, the interspecies *F*_ST_ between *P. mongolica* and *P. meyeri* was greater than the intraspecific *F*_ST_ of *P. meyeri*, suggesting that *P. mongolica* is an independent taxonomic species at the genomic level. This finding is consistent with the results of other studies based on RAPD and ISSR molecular markers and chloroplast genes [17, 18, 20] but differs from the results of morphological comparisons [11–13]. This discrepancy may be due to weak reproductive isolation within the genus *Picea* and frequent gene flow between species leading to phenotypic convergence [40]. Moreover, TreeMix analysis revealed four gene flow events between *P. meyeri* and *P. mongolica*. The ABBA–BABA results revealed significantly more gene flow between *P. koraiensis* and *P. meyeri* than between *P. koraiensis* and *P. mongolica*. This finding is consistent with the greater interspecies *F*_ST_ between *P. mongolica* and *P. koraiensis* than between *P. mongolica* and *P. meyeri*, indicating that *P. mongolica* and *P. meyeri* are closely related. Through structural analysis, we found that when K = 2, which represents the optimal grouping, *P. mongolica* consists of approximately 50% genetic components from *P. koraiensis* and 50% from *P. meyeri*. This suggests that its ancestral type is a hybrid of *P. meyeri* and *P. koraiensis*, corroborating the inference of a reticulate evolutionary history for spruce species [40]. The *P. mongolica* genetic clusters were mixed with the genetic clusters of three *P. meyeri* populations in Hebei. Moreover, the *P. meyeri* populations in Hebei Province exhibited a closer genetic relationship with *P. mongolica* than did the *P. meyeri* populations in Shanxi Province. This suggests that the Hebei *P. meyeri* populations represent transitional populations, consistent with their geographical distribution. The uplift of the Mongolian Plateau at approximately 5 ± 3 Ma created geographical isolation, leading to the differentiation of *P. meyeri* from *P. mongolica* [41]. *P. mongolica* evolved in a relatively isolated geographical environment, while *P. meyeri* migrated southward during the late Pliocene and early Pleistocene when the climate became drastically colder [42], spreading from Hebei to Shanxi. Long-term evolutionary processes and environmental influences shape and preserve genetic variations within populations [43]. Despite the clear geographical differences between *P. meyeri* and *P. mongolica*, frequent gene flow still occurs between them, a phenomenon that is also often observed in other isolated plant lineages [44–46]. Speciation with gene flow may be common in nature [47]. Spatial heterogeneity weakens gene flow, and the divergent selection pressures arising from heterogeneous habitats are a primary driving factor for adaptive speciation [45, 48]. However, during the process of adaptive speciation, gene flow aggregates genetic variations from different populations into a single population, thereby enhancing the genetic variation mediated by selection, which accelerates species diversification [49–51]. Recent research has indicated that moderate gene flow may facilitate adaptive differentiation, primarily because gene flow can reduce the linkage between genetic loci through recombination and segregation, increasing genetic differentiation and thereby promoting speciation [51, 52]. Thus, *P. mongolica* may have originated as a local variety of *P. meyeri* due to geographical isolation and then gradually evolved into an independent species of the *Picea* genus, adapting to sandy environments. However, studies based on chloroplast genes suggest that *P. mongolica* is an ancestor of *P. meyeri* and *P. koraiensis* [20], possibly due to recent radiating differentiation, reticulate evolution, and interspecies plastid recombination in the *Picea* genus [53]. Despite the limited distribution range of *P. mongolica*, this species exhibits greater nucleotide diversity than both *P. meyeri* and *P. koraiensis*, consistent with the findings of previous chloroplast gene-based studies [20]. This high nucleotide diversity enables *P. mongolica* to persist within its narrow distribution range, enhancing its adaptability to sandy environments.

### Signatures of environmental adaptation of the two *Picea* species

Due to their adjacent distributions, *P. meyeri* and *P. mongolica* experienced multiple gene flow events during their differentiation processes [40, 54]. However, due to differences in their ecological environments, they might be strongly influenced by specific environmental factors during evolution, leading to population differentiation at key loci [55, 56]. Subsequently, advantageous mutations may have been preserved through selection, leading to their proliferation and expansion, thereby continuously promoting genetic differentiation within populations, a phenomenon commonly observed in closely related species with adjacent distributions but different ecological niches [57, 58]. Temperature and precipitation are crucial factors affecting species distributions and growth. In this study, IBE and RDA were used to determine the relationships between environmental factors and genetic variation. BIO01, BIO3, BIO12, and BIO15 are important factors explaining the local environmental adaptation of the two *Picea* species. *P. mongolica* occupies the eastern edge of the Hunshandake sandy land, with concentrated stands on both the leeward and windward slopes of the Baiyinaobao sandy land, making it one of the preferred tree species for afforestation in China’s sandy lands [59, 60]. Studies on the *P. mongolica* growth conditions and ecological requirements in sandy lands have shown that *P. mongolica* growth in June accounts for approximately 65% of its annual growth, with the water and thermal conditions in June determining its annual height increase, making seasonal precipitation particularly important for its growth [61]. Conversely, *P. meyeri* is distributed in areas such as Shanxi, Hebei, and parts of Inner Mongolia, where the temperature, rainfall, and humidity are greater than those in the habitats of *P. mongolica*; thus, temperature and annual precipitation significantly impact its distribution. Among the candidate genes related to climate factors, genes associated with plant drought resistance were detected. *BAM1*, *SUS3*, and *CIPK23* can regulate stomatal opening in response to water stress [62–64], while *PUB22* and *PUB23* coordinate the control of drought signaling pathways [65]. *CAT2*, *Sect. 14*, and *ERD4* enhance plant salt stress tolerance [66–68], and *EBF1* and *MPK6* enhance plant cold tolerance [69, 70]. Plants often adapt to abiotic conditions such as climate through positive selection, which leaves traces of selective sweeps on genes linked to those that are selected [2]. Selective sweeps usually reduce genetic diversity within a population but enhance plant environmental adaptability, thereby promoting local adaptation [71]. In the present study, *P. koraiensis* was used as a reference, and genes showing evidence of selective sweeps in *P. meyeri* and *P. mongolica* were related mainly to plant growth and stress resistance. Genes such as *SAHH*, *SPX1*, and *ERF042* were found to be under selection in both species. The *SAHH* gene plays a key role in secondary cell wall biosynthesis in trees [72]. *P. koraiensis*, which is distributed mainly in Northeast China, experiences low winter temperatures, and lignin synthesis in secondary cell walls helps plants resist cold conditions. The *SPX1* gene is closely related to phosphorus homeostasis during plant growth under phosphorus deficiency [73]. *P. mongolica* mainly inhabits sandy habitats where phosphorus is relatively scarce, while *P. meyeri* is mainly distributed in brown forest soil areas with high rainfall and humidity, where phosphorus is easily leached by percolation. *ERF* genes are involved in plant development and stress response regulation. Studies have shown that *ERF042* is related to flower development in angiosperms [74]. The environmental differences between *P. meyeri* and *P. mongolica* have led to differences in their phenological traits. In *P. meyeri*, genes related to plant temperature adaptation, such as *HSP70-6* [75], and those related to pollen development and, in angiosperms, flowering, such as *AMS*, *MYB2*, and *HD3A* [76–78], were also found, reflecting the phenological differences in their distribution areas. In *P. mongolica*, genes related to root growth, such as *ACT2* [79]; drought stress, such as *KPNB1* [80]; and vascular development, such as *ERF018* [81], were found to aid in adaptation to arid environments in sandy areas. With *P. meyeri* as the reference, genes related to stomatal regulation, such as *KIN10A* [82]; drought or cold stress, such as *SRP*, *RGGA*, and *HXK2* [83–85]; and vascular plant seed germination and fruit ripening, such as *PME* [86], were found in *P. mongolica*, indicating that these selectively swept genes enable better adaptation to sandy environments and contributed to its evolution into an independent species within the genus *Picea*. Other researchers have also identified genes related to stomatal regulation, flowering (in angiosperms), and stress resistance among the genes showing evidence of selective sweeps in *P. asperata*, *P. crassifolia*, *P. wilsonii*, and *P. koraiensis* [58, 87, 88]. Throughout history, the habitat of the genus *Picea* has undergone significant changes due to geological events and climate fluctuations [89], resulting in new selection pressures. The process of selective sweeps, which rapidly fixes advantageous genes, has not only enhanced the adaptation of *Picea* species to their environments but has also been a driving force in the diversification of species within the genus [71]. However, the cloning and functional validation of these selected genes remain to be performed.

### Population dynamics and conservation management of two *Picea* species

The stairway plot results indicate that the *N*_e_ values of *P. meyeri* and *P. mongolica* are overall in a state of continuous decline. The *N*_e_ of *P. meyeri* sharply decreased toward the end of the Calabrian period of the Pleistocene due to colder and drier climate conditions, and it likely decreased during the Dali glaciation due to lower temperatures. The *N*_e_ of *P. mongolica* dramatically decreased during the mid-Pleistocene climate transition, which was attributed to the significant increase in aridification [90]. After the species adapted to the arid climate, its *N*_e_ increased, followed by rapid decreases during the early and late phases of the LGM. The SDM results revealed that the total *P. meyeri* and *P. mongolica* suitable habitat continuously decreased from the LGM to the MH and then expanded from the MH to the present. However, *N*_e_ has been continuously decreasing, which may be due to the substantial reduction in *N*_e_ caused by earlier environmental changes. In recent years, human activities have led to continuous reductions in forest resources, especially for *P. mongolica*, which inhabits the ecotone and transition zone of the Hunshandake sandy land, where the pressures from pests and diseases have sharply increased [91, 92]. SDM simulations under future climate change scenarios revealed decreases in the suitable distribution areas for both species, with greater reductions in highly suitable areas and accelerated decreases with increasing greenhouse gas emissions and concentrations. Particularly under high-emission scenarios, *P. mongolica* could lose almost all of its suitable habitat. Notably, *P. mongolica* possesses high genetic diversity and has great potential for adapting to climate change, making it a valuable breeding material. Therefore, *P. mongolica* should be considered an independent species, and conservation measures should be implemented to prevent its extinction. Existing studies indicate that purposeful and conscientious human intervention can promote natural regeneration and the recovery of genetic diversity in natural spruce communities in sandy lands [93]. For *P. mongolica* populations facing the risk of extinction, it is necessary to strengthen the management of water conditions in sandy natural forests to meet their natural regeneration needs. Genetic resource surveys and the collection and management of *P. meyeri* and *P. mongolica* should be conducted, and on this basis, in situ and ex situ conservation areas should be established. Additionally, comprehensive germplasm resource gene banks need to be established to prevent the loss of genetic diversity.

## Conclusion

The GBS of 188 individuals from 13 *P. meyeri* and *P. mongolica* populations revealed genetic differences between the two species. Genomically, although the ancestor of *P. mongolica* may have been a hybrid, *P. mongolica* can be considered an independent species. There have been multiple instances of gene flow between the two species. The *N*_*e*_ of *P. meyeri* decreased from the Early Pleistocene to the present, while that of *P. mongolica* slightly decreased after an initial decline in the Early Pleistocene, followed by a continuous decrease. The total suitable habitat area for both species showed a trend of initially decreasing and then increasing from the previous geological era to the present but is projected to decrease under future climate scenarios. Local adaptation may have facilitated the differentiation of these two species, and selective sweeps revealed candidate genes mainly associated with plant organ development and resistance. The results deepen our understanding of the reticulate speciation mechanisms within the *Picea* genus. Future research could integrate plant phenotypes to explore the mechanisms of interaction between gene function, plant phenotype, and environmental adaptability.

### Supplementary Information

Supplementary Material 1.

## Data Availability

The raw sequencing data generated from this study have been deposited in NCBI SRA (https://www.ncbi.nlm.nih.gov/sra) under the accession number PRJNA876367.

## Abbreviations

GBS: Genotyping-by-sequencing
*N*e: Effective population size
LGM: Last Glacial Maximum
MH: Mid-Holocene
Pun: *P. pungens*
Lik: *P. likiangensis*
Kor1, Kor2: *P. koraiensis* from Mengke mountain and Linjiang, respectively
Mey1, Mey2, Mey3, Mey4, Mey5, Mey6, Mey7, Mey8, Mey9: *P. meyeri* from Saihanba, Kelan, Wuzhai, Shenchi, Ningwu, Jiaocheng, Wutai mountain, XiaoWutai mountain, and Wuling mountain, respectively
Mon1, Mon2, Mon3, Mon4: *P. mongolica* from Baiyinaobao, Dajuzi, Huanggangliang, and Huamugou, respectively
