# Supplementary material for: Adaptive divergence, historical population dynamics, and simulation of suitable distributions for Picea Meyeri and P. Mongolica at the whole-genome level
Source: BMC Plant Biol. 2024 May 30;24:479. doi: 10.1186/s12870-024-05166-6 (PMC11137980; doi:10.1186/s12870-024-05166-6)
Supplement: Supplementary file 1 — Supplementary Material 1. [file 12870_2024_5166_MOESM1_ESM.zip › Fig. S1-S6; Table S1-S2.docx]

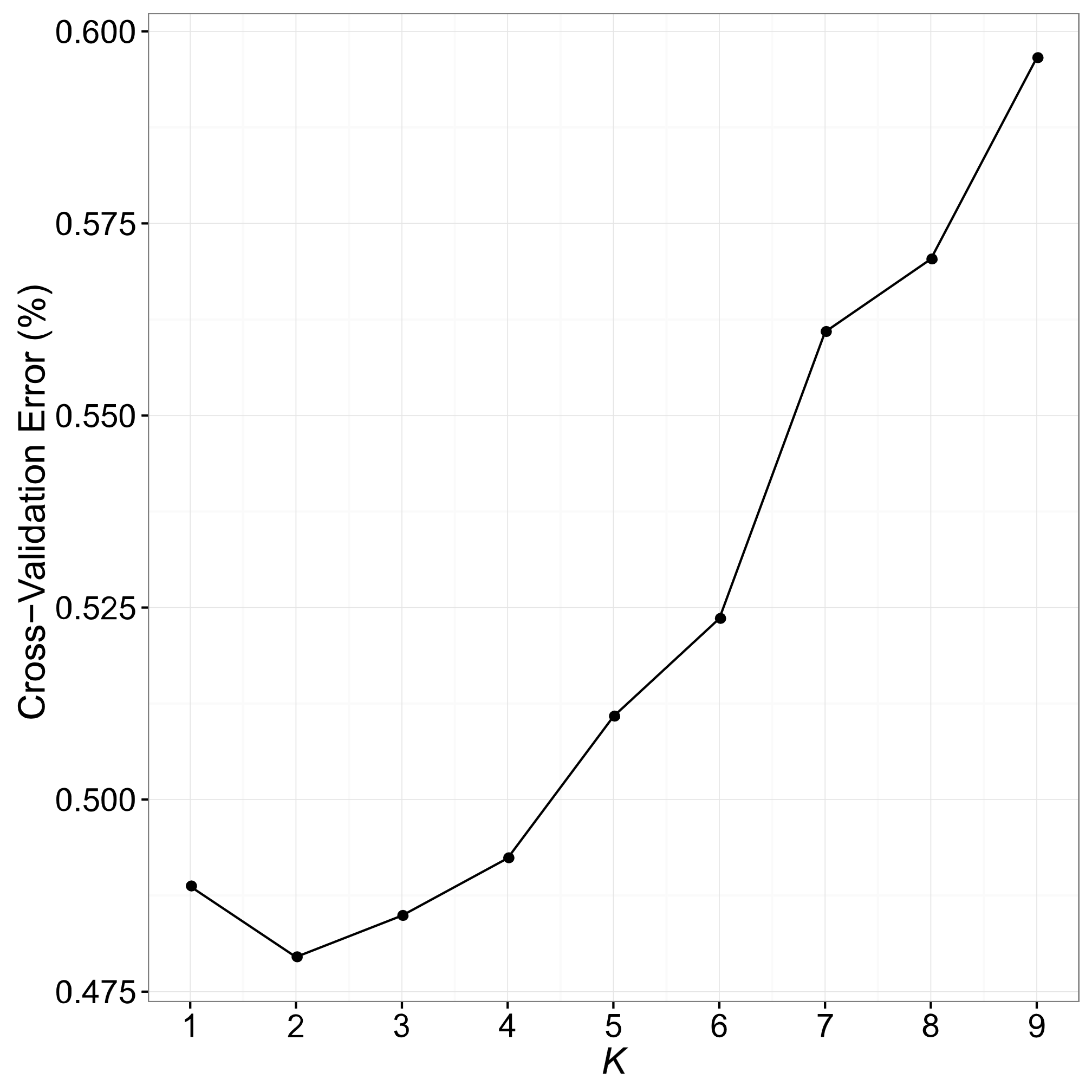


Fig. S1 Cross-validation error value of different populations of spruce based on Admixture


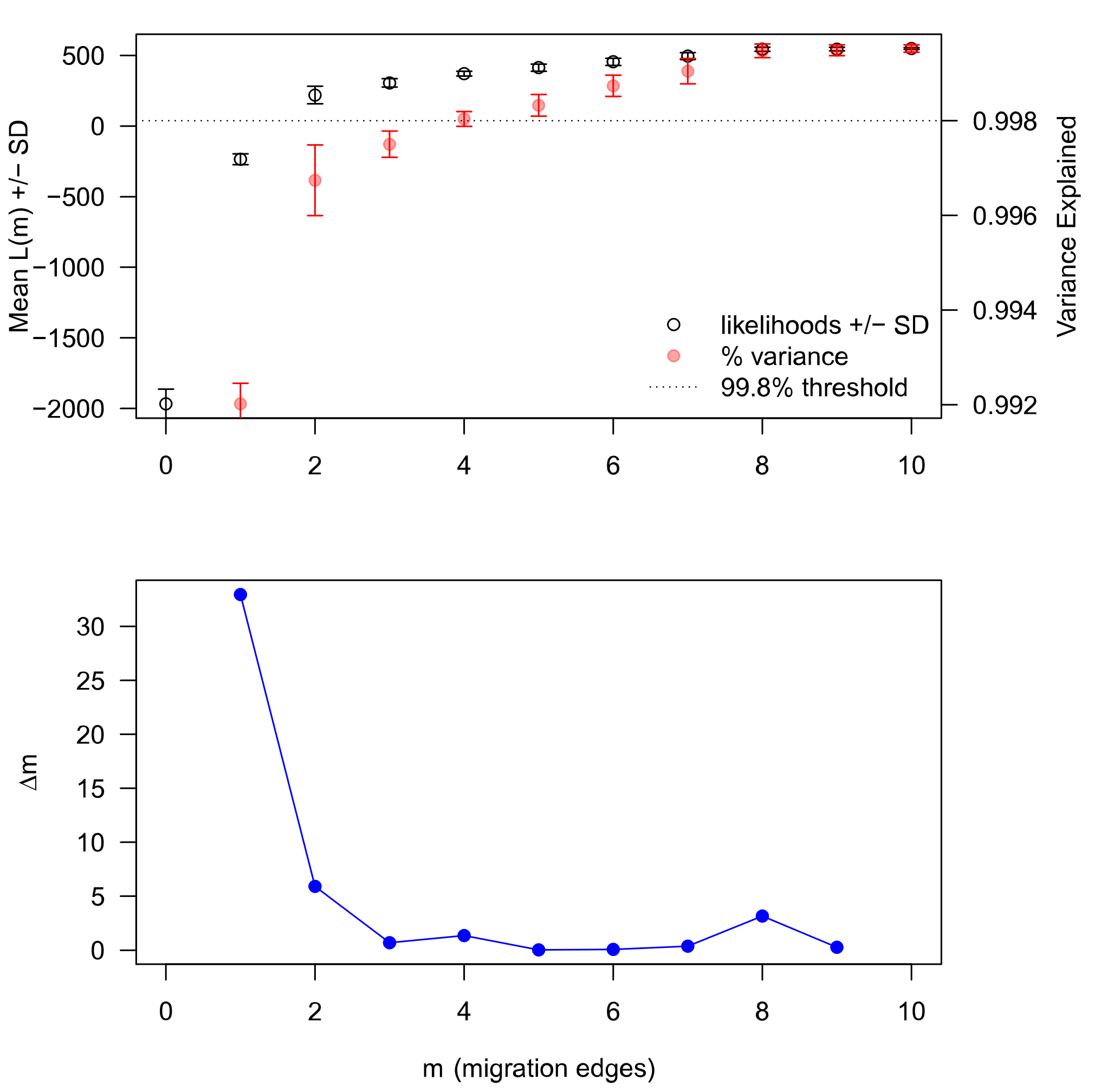


Fig. S2 Optimal number of migration edges


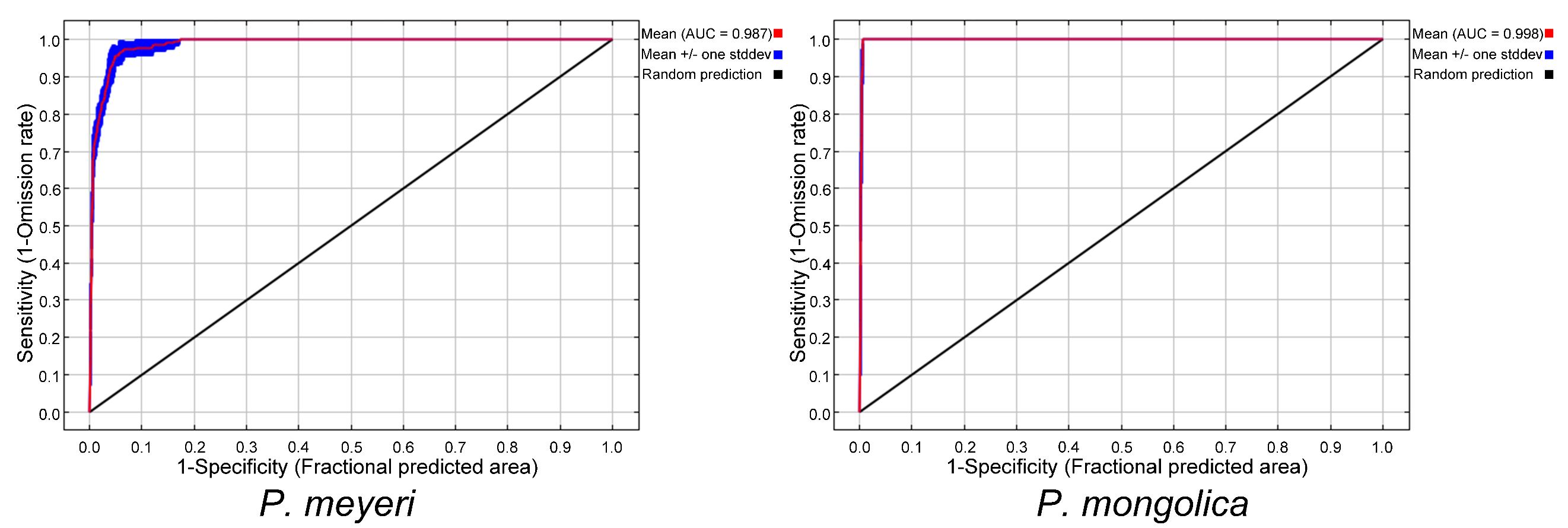


Fig. S3 Receiver operating characteristic curve of spruce MaxEnt models.


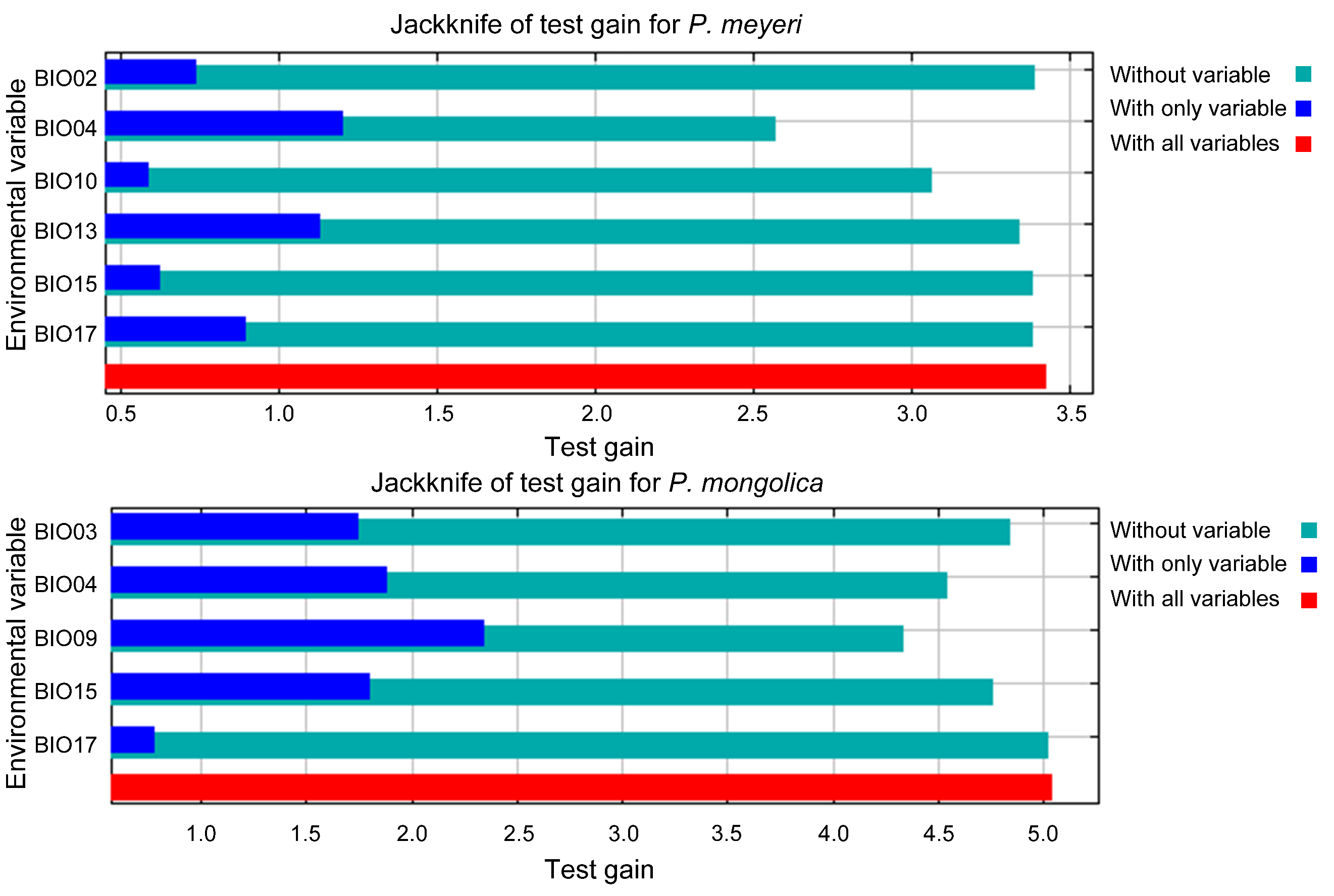


Fig. S4 Evaluation of the relative significance of the leading environmental factors for spruce using the Jackknife of test gain.


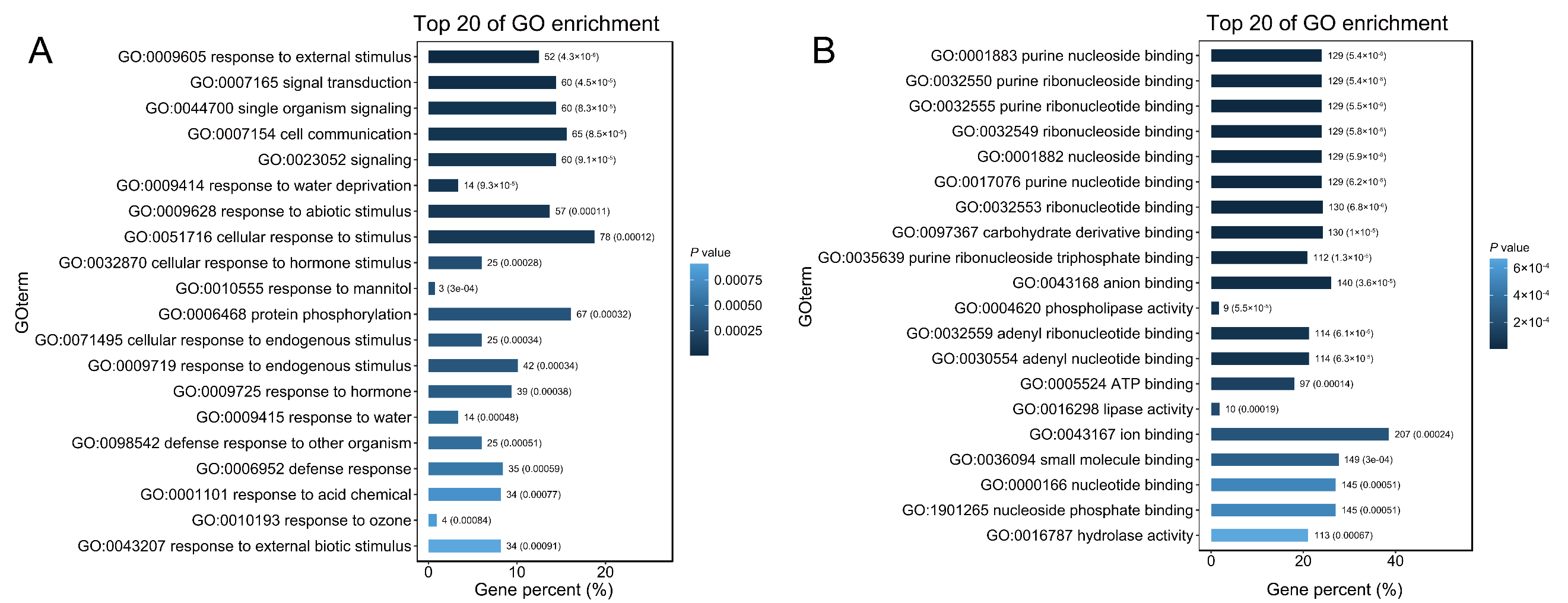


Fig. S5 GO enrichment analysis of potential candidate genes associated with environmental variables. (A) Biological process of candidate genes; (B) Molecular functions of candidate genes.


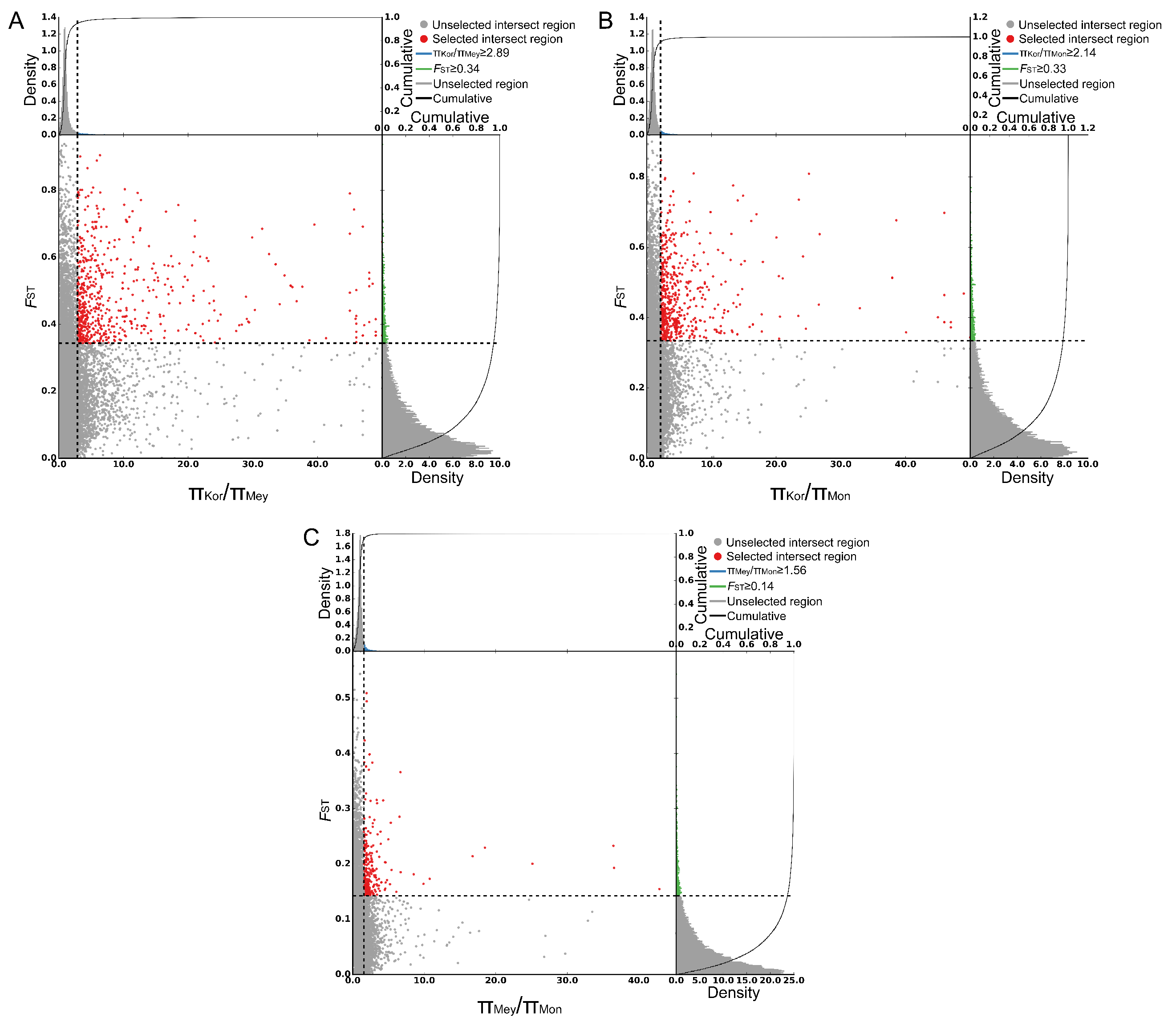


Fig. S6 Regions subject to selective sweeps in *P. meyeri* and *P. mongolica*. (A) Selected areas of *P. meyeri* with *P. likiangensis* as background group; (B) Selected areas of *P. mongolica* with *P. likiangensis* as background group; (C) Selected areas of *P. mongolica* with *P. meyeri* as background group.

Table S1 Geographic information of the spruce populations in the study

| population | species | Sampling location | longitude (E) | latitude (N) | Voucher | BioSample Accession |
| --- | --- | --- | --- | --- | --- | --- |
| Pun | *P. pungens* | Xiaolong mountain, Gansu, China | 106.552853 | 34.475705 | PG392022 | SAMN30656367-30656363 |
| Lik | *P. likiangensis* | Shangri-La, Yunnan, China | 99.811649 | 27.436316 | LG382022 | SAMN30656343-30656347 |
| Kor1 | *P. koraiensis* | Mengke mountain, Heilongjiang, China | 124.309677 | 52.630183 | K022022 | SAMN39300095-39300104 |
| Kor2 | *P. koraiensis* | Linjiang, Jilin, China | 127.387619 | 41.909272 | K122022 | SAMN39300105-39300119 |
| Mey1 | *P. meyeri* | Saihanba, Hebei, China | 117.363309 | 42.395236 | G172022 | SAMN39300020-39300034 |
| Mey2 | *P. meyeri* | Kelan, Shanxi, China | 111.838267 | 38.680916 | G192022 | SAMN39300035-39300049 |
| Mey3 | *P. meyeri* | Wuzhai, Shanxi, China | 111.860519 | 38.795863 | G202022 | SAMN39300050-39300064 |
| Mey4 | *P. meyeri* | Shenchi, Shanxi, China | 112.110307 | 38.966753 | G212022 | SAMN39300065-39300079 |
| Mey5 | *P. meyeri* | Ningwu, Shanxi, China | 111.994779 | 38.867517 | G222022 | SAMN39300080-39300094 |
| Mey6 | *P. meyeri* | Jiaocheng, Shanxi, China | 111.508685 | 37.860432 | G232022 | SAMN35675475-35675461 |
| Mey7 | *P. meyeri* | Wutai mountain, Shanxi, China | 113.599049 | 39.010984 | G242022 | SAMN35675490-35675476 |
| Mey8 | *P. meyeri* | XiaoWutai mountain, Hebei, China | 114.939614 | 39.940559 | G402022 | SAMN35675505-35675491 |
| Mey9 | *P. meyeri* | Wuling mountain, Hebei, China | 117.494444 | 40.595756 | G412022 | SAMN35675520-35675506 |
| Mon1 | *P. mongolica* | Baiyinaobao, Inner Mongolia, China | 117.191181 | 43.518535 | MG092022 | SAMN35675582-35675569 |
| Mon2 | *P. mongolica* | Dajuzi, Inner Mongolia, China | 117.359701 | 42.795127 | MG252022 | SAMN39300120-39300133 |
| Mon3 | *P. mongolica* | Huanggangliang, Inner Mongolia, China | 117.514295 | 43.571249 | MG262022 | SAMN39300134-39300148 |
| Mon4 | *P. mongolica* | Huamugou, Inner Mongolia, China | 117.403294 | 42.664662 | MG272022 | SAMN35675568-35675554 |

Table S2 Latitude and longitude for populations of spruce in the SDM

| *P. meyeri* | | *P. mongolica* | |
| --- | --- | --- | --- |
| longitude (E) | latitude (N) | longitude (E) | latitude (N) |
| 110.4375 | 40.4375 | 116.3125 | 43.2292 |
| 110.7292 | 40.6875 | 116.4792 | 42.8958 |
| 111.4375 | 37.1042 | 116.5208 | 42.8958 |
| 111.4375 | 37.8542 | 116.5208 | 43.5625 |
| 111.4375 | 37.8958 | 116.6042 | 42.6875 |
| 111.4792 | 37.6458 | 116.6458 | 42.5208 |
| 111.4792 | 37.6875 | 116.6875 | 42.8542 |
| 111.4792 | 37.7292 | 116.7708 | 43.6458 |
| 111.4792 | 37.7708 | 116.8542 | 42.8958 |
| 111.4792 | 37.8542 | 116.8542 | 43.6875 |
| 111.5625 | 37.8542 | 116.8958 | 43.6875 |
| 111.5625 | 37.8958 | 117.1458 | 43.3125 |
| 111.6042 | 37.8958 | 117.1875 | 43.3542 |
| 111.6875 | 38.3542 | 117.1875 | 43.5208 |
| 111.7292 | 38.6458 | 117.1875 | 43.5625 |
| 111.7708 | 38.6875 | 117.1875 | 43.6042 |
| 111.7708 | 39.0208 | 117.2292 | 42.5625 |
| 111.7708 | 41.1042 | 117.2292 | 42.8125 |
| 111.8125 | 38.7708 | 117.2292 | 43.4792 |
| 111.8542 | 38.8125 | 117.2292 | 43.5208 |
| 111.8958 | 38.7292 | 117.2292 | 43.5625 |
| 111.9375 | 38.7292 | 117.2292 | 43.8542 |
| 111.9375 | 38.8125 | 117.2708 | 43.5625 |
| 111.9375 | 38.8542 | 117.3125 | 43.8958 |
| 111.9375 | 38.8958 | 117.3542 | 43.8125 |
| 111.9792 | 38.8542 | 117.3542 | 43.8542 |
| 112.0208 | 38.7292 | 117.3958 | 42.6458 |
| 112.0208 | 38.8542 | 117.3958 | 43.4375 |
| 112.0625 | 38.9792 | 117.4375 | 43.3125 |
| 112.1042 | 38.8125 | 117.5208 | 43.6042 |
| 112.1042 | 38.8958 | 117.6458 | 43.7708 |
| 112.1042 | 38.9375 |  |  |
| 112.1042 | 38.9792 |  |  |
| 112.3125 | 38.9792 |  |  |
| 112.3125 | 40.6042 |  |  |
| 113.4375 | 39.7708 |  |  |
| 113.4792 | 39.0625 |  |  |
| 113.5625 | 39.0208 |  |  |
| 113.6042 | 39.0208 |  |  |
| 113.6042 | 39.9375 |  |  |
| 113.6042 | 39.9792 |  |  |
| 113.6042 | 40.0625 |  |  |
| 113.6458 | 39.0625 |  |  |
| 113.6458 | 39.1458 |  |  |
| 113.8958 | 38.9375 |  |  |
| 114.8958 | 40.7708 |  |  |
| 114.9375 | 39.8542 |  |  |
| 114.9375 | 39.9375 |  |  |
| 114.9792 | 39.8125 |  |  |
| 114.9792 | 39.8542 |  |  |
| 114.9792 | 39.9375 |  |  |
| 115.0208 | 39.8542 |  |  |
| 115.0208 | 39.8958 |  |  |
| 115.0625 | 39.9375 |  |  |
| 115.0625 | 39.9792 |  |  |
| 115.0625 | 40.0208 |  |  |
| 115.1042 | 40.4792 |  |  |
| 115.1875 | 39.7708 |  |  |
| 115.2292 | 39.8125 |  |  |
| 115.2292 | 40.0625 |  |  |
| 115.2708 | 39.3125 |  |  |
| 115.3542 | 39.3542 |  |  |
| 115.4375 | 39.3542 |  |  |
| 115.4792 | 40.0625 |  |  |
| 115.6042 | 39.8542 |  |  |
| 115.8125 | 42.8542 |  |  |
| 116.3125 | 43.2292 |  |  |
| 116.5208 | 42.8958 |  |  |
| 116.6458 | 42.5208 |  |  |
| 116.8542 | 42.8125 |  |  |
| 116.8958 | 42.4375 |  |  |
| 116.9792 | 42.4375 |  |  |
| 117.2292 | 42.3958 |  |  |
| 117.3542 | 42.4375 |  |  |
| 117.4792 | 40.6042 |  |  |
| 118.3125 | 43.9375 |  |  |
| 118.4375 | 41.3958 |  |  |
